# Cultivation and preliminary chemical analyses of the sclerotium-forming, termite fungus garden-associated *Xylaria minima*

**DOI:** 10.3897/mycokeys.139.206447

**Published:** 2026-08-21

**Authors:** Er-Fu Yang, Marc Stadler, Anton Möllerke, Milan C. Samarakoon, Chun-I Chiu, Dong-Qin Dai, Itthayakorn Promputtha, Fu-Qiang Yu, Sajad Ali, Samantha C. Karunarathna, Saowaluck Tibpromma, Jing-Ya Yang

**Affiliations:** 1 Center for Yunnan Plateau Biological Resources Protection and Utilization, College of Biology and Food Engineering, Qujing Normal University, Qujing 655099, China Institute of Microbiology, Technische Universität Braunschweig Braunschweig Germany; 2 Department of Biology, Faculty of Science, Chiang Mai University, Chiang Mai 50200, Thailand Center for Yunnan Plateau Biological Resources Protection and Utilization, College of Biology and Food Engineering, Qujing Normal University Qujing China; 3 Helmholtz Centre for Infection Research (HZI), Department Microbial Drugs, 38124 Braunschweig, Germany Key Laboratory of Yunnan Provincial Department of Education of the Deep-Time Evolution on Biodiversity from the Origin of the Pearl River, Qujing Normal University Qujing China; 4 Institute of Microbiology, Technische Universität Braunschweig, Spielmannstraße 7, 38106 Braunschweig, Germany Yunnan Key Laboratory for Fungal Diversity and Green Development & Yunnan International Joint Laboratory of Fungal Sustainable Utilization in South and Southeast Asia, Germplasm Bank of Wild Species, Kunming Institute of Botany, Chinese Academy of Sciences Kunming China; 5 Department of Entomology and Plant Pathology, Faculty of Agriculture, Chiang Mai University, Chiang Mai 50200, Thailand Helmholtz Centre for Infection Research (HZI), Department Microbial Drugs Braunschweig Germany; 6 Key Laboratory of Yunnan Provincial Department of Education of the Deep-Time Evolution on Biodiversity from the Origin of the Pearl River, Qujing Normal University, Qujing, Yunnan Province, 655011, China School of Agronomy and Biological Science, Yuxi Normal University Yuxi China; 7 Yunnan Key Laboratory for Fungal Diversity and Green Development & Yunnan International Joint Laboratory of Fungal Sustainable Utilization in South and Southeast Asia, Germplasm Bank of Wild Species, Kunming Institute of Botany, Chinese Academy of Sciences, Kunming, Yunnan Province 650201, China Department of Biology, Faculty of Science, Chiang Mai University Chiang Mai Thailand; 8 Department of Biological Sciences, College of Science, King Faisal University, Al-Ahsa 31982, Saudi Arabia Department of Entomology and Plant Pathology, Faculty of Agriculture, Chiang Mai University Chiang Mai Thailand; 9 School of Agronomy and Biological Science, Yuxi Normal University, Yuxi 653100, China Department of Biological Sciences, College of Science, King Faisal University Al-Ahsa Saudi Arabia

**Keywords:** *Macrotermes* spp., small sclerotia, termite fungus comb, Wulingshen, xylariaceous fungi

## Abstract

*Xylaria nigripes* and its close relatives, specialized for their associations with termite fungus combs, are capable of producing massive sclerotia within the chambers of dead termite nests. These sclerotia, traditionally referred to in Chinese medicine as “Wulingshen,” exhibit a wide range of pharmacological activities. Massive *Xylaria* sclerotia are typically harvested from natural habitats, and their formation under laboratory conditions has rarely been reported. This study induced the formation of small sclerotia in *Xylaria minima* by incubating mature *Macrotermes* fungus combs. Sclerotium formation on the comb was characterized by an inconspicuous vegetative growth phase, rapid sclerotial development, early dormancy, and temporally delayed reproduction. Sclerotia formed more sporadically on potato dextrose agar (PDA), water agar (WA), and oat-based media. In contrast, growth on comb leachate agar (CLA) was faster, and sclerotia developed readily. The sclerotia of *X. minima* show strong potential for cultivation using comb materials and agricultural substrates. Exploratory chemical analyses revealed that sclerotia were enriched in crude polysaccharides, alkaloids, and terpenoids, as well as amino acids and amino acid derivatives such as L-glutamic acid and γ-aminobutyric acid (GABA), compared with mycelia. These findings indicate that *X. minima* does not exhibit strict substrate specificity for sclerotium formation and highlight its potential for future studies on cultivation and bioactive compound discovery. This study additionally highlights that the sclerotium-forming habit in comb-associated *Xylaria* species has likely evolved independently multiple times, occurring in both the newly reported small-sclerotium-forming *X. minima* and the *X. nigripes* species complex.

## Introduction

In fungus-farming termite systems, certain members of the genus *Xylaria* have evolved a “sit-and-wait” strategy to evade termite hygienic behaviors. Once a fungus garden is compromised or abandoned, *Xylaria* species rapidly colonize the ecological niche formerly occupied by termite-cultivated *Termitomyces* species (Fricke et al. 2023; Agarwal et al. 2024; Yang et al. 2025). This clade of *Xylaria* species is primarily associated with termite nests (the TE clade) and forms a distinct, phylogenetically defined clade within *Xylaria* that contains approximately 45 described taxa (Ju and Hsieh 2007; Hsieh et al. 2020; Li et al. 2025; Okane et al. 2025). As the TE clade is almost exclusively found in the fungus gardens (combs) of fungus-farming termites in the subfamily *Macrotermitinae*, this lineage has historically been considered the subgenus *Pseudoxylaria* (Hsieh et al. 2010; Wangsawat et al. 2021). However, as *Pseudoxylaria* is not recognized as a formal taxonomic group, its members are generally referred to as either *Xylaria* of the TE clade or termite-associated *Xylaria* (Li et al. 2025). Earlier studies failed to detect *Xylaria* in the surrounding soil of nests (Thomas 1987), and current evidence suggests that *Xylaria* is most likely introduced through termite foraging activities and has coevolved with its termite hosts and comb substrates to occupy a unique ecological niche for survival (Visser et al. 2009; Wangsawat et al. 2021; Fricke et al. 2023; Li et al. 2025). Compared with free-living *Xylaria* species, termite-associated *Xylaria* exhibit adaptations to comb-specific stressors, including reduced genome size, decreased protein-coding gene content, loss of functional capacities, and production of novel metabolites associated with antimicrobial and insect antifeedant activities, which together may reduce antagonistic interactions (Fricke et al. 2023). Termite-associated *Xylaria* species are not restricted to association with a single termite species (Visser et al. 2009; Li et al. 2025). Single-gene phylogenies provide no evidence for host specificity (Guedegbe et al. 2009; Visser et al. 2009; Hsieh et al. 2010); therefore, a higher-resolution phylogeny is required to resolve the extent of host specificity (Li et al. 2025).

Approximately 45 comb-specialized *Xylaria* species have so far been described (Okane et al. 2025), but not all of their stromata or sclerotia can be artificially induced to grow from fungus combs that are no longer tended by termites. Some researchers have speculated that, due to microbial interactions, competition, and termite hygiene behaviors, *Xylaria* is not present throughout the comb as a continuous mycelium but rather as spores or small mycelial patches (Batra and Batra 1979; Visser et al. 2009). In addition, termite-associated *Xylaria* species can produce massive sclerotia, a trait currently known only from the clade including *X. nigripes* and its close relatives (e.g., *Xylaria acuminatilongissima*, *X. mianyangensis*, *X. neonigripes*, *X. rogersionigripes*, and *X. subescharoidea*); multilocus phylogenetic analyses suggested that the sclerotium-forming habit arose only once within *Xylaria* (Ju et al. 2022; Hsieh and Ju 2024). *Xylaria* sclerotia are resistant structures composed of tightly compacted hyphae; those formed within subterranean termite nests are massive compared with those produced by other fungi (Hsieh and Ju 2024), vary in shape from spherical to ovoid or even irregular, and are several centimeters in diameter. These sclerotia gradually shrink until they disappear completely after the nutrients are depleted (Ju et al. 2022; Hsieh and Ju 2024).

Massive *Xylaria* sclerotia have been consumed as traditional herbal medicine in China and are referred to as “Wulingshen”; they are believed to be effective in eliminating internal dampness, alleviating infantile convulsions, relieving palpitations, promoting lactation, nourishing the heart and kidneys, and treating insomnia, nosebleeds, and postpartum hemorrhage (Ying et al. 1987; Lin et al. 2013; Ju et al. 2022; Hsieh and Ju 2024). Contemporary pharmacological investigations of the massive sclerotium-forming *Xylaria nigripes* have substantiated its traditional medicinal applications and revealed a broad array of pharmacological activities. For example, several newly identified metabolites (xylarinaps A–E and xylaribrasilaids A–D) from *X. nigripes* have been reported to exhibit *in vitro* neuroprotective activity in PC12 cells and may exert their effects through antioxidative and antiapoptotic mechanisms (Li et al. 2021; Long et al. 2025). A randomized, double-blind, placebo-controlled pilot clinical trial showed that *X. nigripes* could alleviate depressive symptoms in patients with epilepsy (Peng et al. 2015). Xn-40, a polysaccharide fraction from *X. nigripes* obtained by 40% ethanol precipitation showed the strongest *in vitro* antioxidant activity and significantly attenuated reactive oxygen species (ROS) generation in HaCaT cells without cytotoxicity, suggesting its potential as a natural antioxidant for skin protection (Chang et al. 2018). Crude extracts of *X. nigripes* have been reported to exhibit strong anti-inflammatory and immunomodulatory activities in *in vitro* (RAW264.7 cells) and *in vivo* (BALB/c mice) models, suppressing pro-inflammatory mediators while enhancing IL-10 production, splenocyte proliferation, and natural killer (NK) cell activity without observable toxicity (Divate and Chung 2017). In addition, other studies have demonstrated that polysaccharide fractions from *X. nigripes* possess significant anti-inflammatory activity *in vitro* (Jen et al. 2021). However, sclerotium-forming *Xylaria* species other than *X. nigripes* have rarely been reported, and pharmacological investigations and studies on sclerotium induction, cultivation, and toxicity remain limited. The underlying mechanisms and ecological significance of sclerotium formation remain unresolved (Hsieh and Ju 2024). Therefore, the bioactive compounds present in mycelia and sclerotia warrant further chemical and pharmacological investigation, as well as further investigation into their sclerotium-forming mechanism and cultivation potential.

In this study, six colonies of *Macrotermes annandalei* and *M. gilvus* were sampled in Xishuangbanna, China. The purpose of this study was to investigate the feasibility of inducing and isolating *Xylaria* sclerotia and the cultivability of sclerotia under controlled laboratory conditions. A total of 600 comb fragments (2 g each) from fresh and mature combs were used to determine the capacity of combs to produce sclerotia. Sclerotium-forming *Xylaria* was isolated, followed by a series of media-based and substrate-based cultivation experiments. In parallel, preliminary chemical analyses were performed on cultures grown on potato dextrose agar (PDA) and their artificially grown sclerotia. For this purpose, quantitative analyses of chemical constituents, including three major metabolite classes (polysaccharides, alkaloids, and triterpenoids/sterols), were performed using traditional phytochemical methods. In addition, the occurrence of selected amino acids and their derivatives (L-glutamic acid and γ-aminobutyric acid) was surveyed in the mycelia and sclerotia of sclerotium-forming *Xylaria*.

## Methods

### Sample collection, sclerotia formation, and fungal isolation

**Figure 1.**
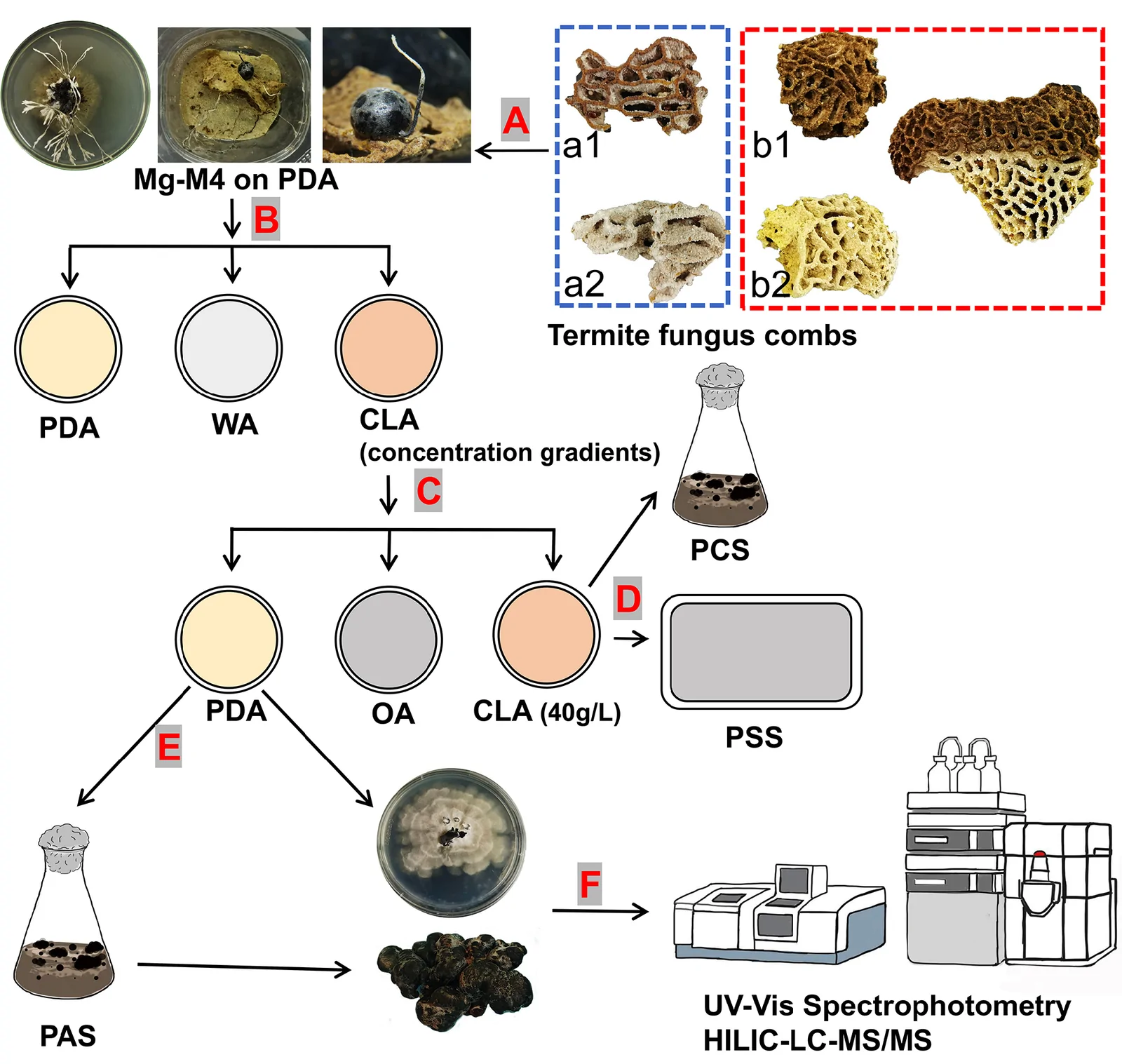
**A**. *Xylaria* sclerotia induced from 2 g termite fungus combs: fresh combs of *M. annandalei* (a1) and *M. gilvus* (b1) and mature combs of *M. annandalei* (a2) and *M. gilvus* (b2); **B**. Strains isolated from sclerotial tissues of Mg-M4 growing on PDA were transferred onto fresh PDA, WA, and CLA media at different concentrations; **C**. *Xylaria* mycelia derived from 40 g/L CLA and subsequently subcultured on PDA, OA, and CLA (40 g/L); **D**. Mycelial plugs grown on CLA inoculated onto paper-based comb substrates (PCS) and paper-based soil substrates (PSS); **E**. Mycelial plugs grown on PDA inoculated onto paper-based agricultural substrates (PAS); **F**. Fresh, pure mycelia collected from PDA and sclerotia harvested from PAS were sent to a commercial service provider for chemical analyses using UV–Vis spectrophotometry and HILIC–LC–MS/MS.

Three colonies of *M. annandalei* and three colonies of *M. gilvus* were investigated in Xishuangbanna, China (21°41'N, 101°25'E, 800 m) in January 2025. From each colony, termite fungus combs were carefully excavated and individually transferred to sterile plastic containers (20 × 10 × 6 cm) that had been disinfected with 75% ethanol and exposed to UV light for 30 min before use, and the samples were transported to the laboratory at Qujing Normal University (China). Fungus combs were incubated to induce the development of *Xylaria* species according to previously published methods (Visser et al. 2009; Fricke et al. 2023), with slight modifications. Fifty fresh fungus comb (FC) fragments and 50 mature comb (MC) fragments, each weighing 2 g, were collected from each termite colony, yielding a total of 300 fresh comb fragments and 300 mature comb fragments. Each sample was placed individually in a sterile plastic box (4 × 3 × 5 cm) that had been disinfected with 75% ethanol and exposed to UV light for 30 min before use and was lined with a layer of sterile, moistened tissue paper to maintain humidity. All samples were incubated at approximately 28 °C in the dark. After 15 days, the number of boxes containing sclerotia was recorded (Fig. 1A). Long-term continuous observations of termite comb fragments were then conducted, and sclerotium-forming pieces were monitored daily without opening the boxes until germinating hyphae emerged through the sclerotial husk. Finally, all visible sclerotia were harvested, and the wet weight and diameter of each sclerotium were measured. Fresh sclerotia were then surface-disinfected with 75% ethanol and dissected aseptically, and the internal tissues were transferred to PDA plates under a laminar flow hood (Fig. 1B). Living cultures obtained from each sclerotium were deposited in the Guizhou Medical University Culture Collection (GMBCC), Guiyang, China.

### DNA extraction, PCR amplification, and sequencing

Pure fungal mycelia were harvested from PDA and transferred to sterile 1.5 mL microcentrifuge tubes for genomic DNA extraction. Genomic DNA was extracted using the Biospin Fungus Genomic DNA Extraction Kit (BioFlux®, Hangzhou, China) following the manufacturer’s protocol. PCR amplifications were performed in a total reaction volume of 25 μL containing 8.5 μL of double-distilled water, 12.5 μL of 2× Power Taq PCR MasterMix, 2 μL of DNA template, and 1 μL each of the forward and reverse primers (10 pmol). The internal transcribed spacer (ITS) region was amplified using the primer pair ITS4 and ITS5 (White et al. 1990), the α-actin gene (*ACT*) was amplified using the primer pair 512F and 738R (Carbone and Kohn 1999), β-tubulin 2 (*TUB2*) was amplified using the primer pair T1 and T22 (O’Donnell and Cigelnik 1997), and the DNA-dependent RNA polymerase II largest subunit (*RPB2*) was amplified using the primer pair 5F and 7cR (Liu et al. 1999). The PCR cycling conditions consisted of an initial denaturation at 94 °C for 4 min, followed by 30 cycles of 94 °C for 30 s, annealing at gene-specific temperatures (Suppl. material 2: table SS1), and extension at 72 °C for 1 min, with a final extension at 72 °C for 10 min. PCR products were sequenced by Sangon Biotech Co., Ltd. (Shanghai, China). All newly generated sequences were deposited in GenBank, and accession numbers are provided in Suppl. material 2: tables S2, S3.

### Phylogenetic analyses

Forward and reverse sequences were assembled and edited using Geneious version 9.1.2 (https://www.geneious.com, accessed 12 May 2026). A total of 87 *Xylaria* strains representing the TE clade, together with their corresponding GenBank accession numbers, are listed in Suppl. material 2: table SS2. Multiple sequence alignments were generated using the MAFFT online server (www.ebi.ac.uk/Tools/mafft, accessed on 12 May 2026) (Katoh and Standley 2013) and manually adjusted in BioEdit version 7.2.3 (Hall 1999) when necessary. Ambiguous and uninformative regions of sequences were trimmed using trimAL version 1.2 (http://trimal.cgenomics.org, accessed on 12 May 2026), and alignments of different gene regions (ITS, *ACT*, *TUB2*, and *RPB2*) were concatenated in BioEdit. The Alignment Transformation Environment (ALTER) (Glez-Peña et al. 2010) was used to convert FASTA files to PHYLIP format for maximum likelihood (ML) analyses and to NEXUS format for Bayesian inference (BI) analyses. The ML analyses were conducted in the CIPRES Science Gateway version 3.3 (http://www.phylo.org/portal2, accessed 12 May 2026) (Miller et al. 2010), using RAxML-HPC2 on XSEDE (version 8.2.12) (Stamatakis 2014) with 1,000 bootstrap iterations under the GTRGAMMA substitution model. Bayesian inference (BI) analyses were performed in MrBayes on XSEDE (version 3.2.7a) via the same CIPRES platform (Ronquist et al. 2012), applying the best-fit evolutionary model determined by MrModeltest version 2.3 (Nylander et al. 2008) and PAUP version 4.0b10 (Swofford 2003). The GTR+I+G model was selected for BI analyses. Six Markov chains were run simultaneously for 10,000,000 generations, with samples and trees generated every 1,000 generations. Bayesian posterior probabilities (BYPP) (Zhaxybayeva and Gogarten 2002; Erixon et al. 2003) were calculated from the resulting MCMC samples. The resulting multigene phylogenetic trees were visualized in FigTree version 1.4.0 (http://tree.bio.ed.ac.uk/software/figtree/, accessed 30 May 2026) and finalized in Microsoft PowerPoint. BI posterior probabilities and ML bootstrap support values equal to or greater than 0.95 and 70%, respectively, are shown at the first and second positions above the nodes.

### Estimation of growth of sclerotium-forming *Xylaria* fungi on termite-comb leachate agar at different leachate concentrations

In the sclerotium formation experiment on media and substrates, *Xylaria* sp. strain Mg-M4, originally isolated from the largest sclerotia, was selected for all subsequent assays (see Results). Experiments were conducted to evaluate the effects of termite-comb leachate concentration in water agar (WA) on radial growth and sclerotium formation in strain Mg-M4 and to compare fungal growth on WA and PDA (Fig. 1B). Media were prepared as follows: WA with 15 g/L agar, PDA at 39 g/L, and comb leachate agar (CLA) by adding leachate to WA at various concentrations (all media from HiMedia Laboratories, Mumbai, India) (Fig. 1B). Mature fungus combs of *M. gilvus* were collected from colonies in Xishuangbanna and transported to the laboratory. The garden material was pooled, finely crushed under sterile conditions, and subjected to three freeze–thaw cycles to suppress microbial activity (Haei et al. 2011). The frozen material was then oven-dried at 60 °C and weighed using a high-precision balance.

To prepare CLA, dried comb material (5, 10, 20, 40, 80, 160, and 320 g) was added separately to 1 L of distilled water and incubated at 60 °C for 2 h with gentle shaking. During incubation, the mixtures were intermittently manually agitated to facilitate the release of soluble compounds from the comb material. The resulting suspensions were filtered three times through sterile cheesecloth until only liquid remained, and the final volume was adjusted to 1 L with distilled water. The pH of the leachates ranged from 4.49 to 4.69 across concentrations and was not adjusted (measured using an OHAUS benchtop pH meter). WA (HiMedia, 18 g/L) was prepared using each termite-comb leachate as the solvent, yielding seven leachate concentrations expressed as weight/volume (w/v): 0 g/L (distilled water control), 10 g/L, 20 g/L, 40 g/L, 80 g/L, 160 g/L, and 320 g/L. All media were autoclaved at 121 °C for 20 min and poured into sterile Petri dishes (90 mm diameter; 20 mL per plate). WA (HiMedia, 18 g/L) and PDA (HiMedia, 39 g/L) plates were prepared in parallel and served as conventional control media.

For *Xylaria* sp. strain Mg-M4, three replicate plates were prepared for each leachate concentration and for the control media (WA and PDA). A 5 mm-diameter mycelial plug taken from the margin of a 1-month-old PDA culture was placed at the center of each 90 mm Petri dish (three replicates). All cultures were incubated in darkness at room temperature, and radial mycelial growth was measured after 15 days. These data were used to assess the effects of termite-comb leachate concentration and culture medium on the growth of strain Mg-M4.

### Observation of sclerotium formation on different media and substrates

To evaluate whether sclerotium formation and vegetative growth of strain Mg-M4 exhibit specificity for comb material, an experiment comprising three components (Parts A–C) was designed. These were (A) plate-based assays on standard laboratory media, (B) flask incubations using autoclaved termite comb material, and (C) substrate tests using agricultural substrates and field soil.

Part A comprised plate-based tests in 90 mm dishes using PDA, CLA, and oat media. Strain Mg-M4 (1-month-old culture on CLA [40 g/L]) was transferred to PDA (HiMedia, 39 g/L) and oat agar (HiMedia; oatmeal, 60 g/L; agar, 15 g/L) plates. Plates were incubated in darkness at room temperature to observe whether sclerotium formation occurred (Fig. 1C).

Part B involved a mature comb substrate. A 20 g portion (dry weight) of mature comb material from *M. gilvus* was mixed with pieces of sterile tissue paper and 40 mL of distilled water (to obtain ~60–70% moisture content), placed into sterile 250 mL Erlenmeyer flasks (three replicates), and autoclaved three times at 121 °C for 20–30 min. After cooling to room temperature, five mycelial plugs (5 mm diameter) of Mg-M4 grown on 40 g/L CLA were aseptically inoculated into each flask. Erlenmeyer flasks were incubated in the dark at room temperature for 1 month, and observations were made to determine whether sclerotia formed (Fig. 1D).

Part C used an agricultural-waste substrate. A laboratory substrate was prepared by mixing, for a total dry weight of 20 g, wheat grain (3 g), corn flour (3 g), sawdust (6 g), cottonseed hull (5 g), wheat bran (3 g), and pieces of sterile tissue paper. Distilled water (40 mL) was added to reach ~60–70% moisture, and the substrate was mixed thoroughly and transferred to 250 mL Erlenmeyer flasks (four replicates) (Fig. 1E). Flasks containing the substrate were autoclaved twice at 121 °C for 20–30 min. After cooling, five mycelial plugs (5 mm diameter) of Mg-M4 (from PDA [see Fig. 1E]) were aseptically placed on the substrate surface in each flask. Flasks were incubated in the dark at room temperature for 1 month, after which sclerotia, if present, were collected. To test soil as a substrate, 20 g of dry soil (collected from around the termite mound) was mixed with 40 mL of distilled water and several pieces of sterile tissue paper. The mixture was then placed into sterile square boxes (13 × 10 × 4.2 cm; three replicates). After autoclaving three times at 121 °C for 20–30 min, half of a CLA plate (40 g/L; 90 mm diameter) colonized with Mg-M4 was placed on the soil surface to promote rapid colonization, and the boxes were covered with sterile aluminum foil. After 1 month in darkness at room temperature, the boxes were opened aseptically and inspected for sclerotia. Any sclerotia formed were observed and collected (Fig. 1D).

For each substrate and replicate, sclerotium formation was recorded as present or absent after incubation, and the maximum sclerotial size was documented for comparison (see Results). When sclerotium-forming *Xylaria* sporulated on the media or substrates, micromorphological features were documented with a digital camera (Canon EOS 600D, Canon Inc., Tokyo, Japan) mounted on a compound microscope (Nikon ECLIPSE Ni, Nikon, Tokyo, Japan). The dimensions of conidia were measured using the Tarosoft® Image Frame Work (IFW) software. Color images were assembled and processed in Adobe Photoshop CS3 Extended version 10.0 (Adobe®, San Jose, CA, USA) to produce complete photo plates.

### Preliminary analytical characterization of sclerotia and mycelia

Sclerotia were harvested from paper-based agricultural substrate (PAS), while mycelial biomass was obtained from PDA cultures. For analysis, fresh sclerotia (10 g) and fresh mycelial biomass (10 g) were sent to Sangon Biotech Co., Ltd. (Shanghai, China) (Fig. 1F).

Crude polysaccharides, total triterpenoids, total sterols, and total alkaloids were determined using standard colorimetric assays as described in previous reports (DuBois et al. 1956; Xing et al. 2002; Sabir et al. 2003; Pedrosa et al. 2020). These assays were applied for preliminary, semiquantitative chemical profiling of sclerotia and mycelia of *Xylaria* sp. strain Mg-M4, without biological replication.

Samples were extracted using appropriate solvent systems specific to each metabolite class: water for crude polysaccharides, ethanol for total triterpenoids, chloroform for total sterols, and methanol for total alkaloids. All extractions were performed under ultrasound-assisted conditions, followed by centrifugation (5000 × *g*, 10 min) and filtration.

Quantification was performed using a colorimetric method with UV–Vis spectrophotometry (Agilent 8453, Santa Clara, CA, USA) at 490 nm (polysaccharides), 548 nm (triterpenoids), 543 nm (sterols), and 414 nm (alkaloids), respectively. Glucose, oleanolic acid, β-sitosterol, and matrine (Gan et al. 2017; Lin et al. 2019) were used as external standards. The contents of crude polysaccharides, total triterpenoids, total sterols, and total alkaloids were expressed as glucose, oleanolic acid, β-sitosterol, and matrine equivalents (g/100 g fresh weight), respectively.

### HILIC–LC–MS/MS analysis of glutamate and γ-aminobutyric acid (GABA)

L-glutamic acid and γ-aminobutyric acid (GABA) are the principal excitatory and inhibitory neurotransmitters, respectively, in the mammalian central nervous system, where they regulate neuronal excitability by modulating synaptic transmission (Zhou and Danbolt 2014; Barakat and Aljutaily 2025). Both compounds have been detected in the Wuling capsule™, a drug for insomnia derived from *X. nigripes* (Chen et al. 1990; He and Liu 2010). Therefore, they were determined using hydrophilic interaction liquid chromatography coupled with tandem mass spectrometry (HILIC–LC–MS/MS), following established procedures with minor modifications (Eckstein et al. 2008; Buck et al. 2009; Inoue et al. 2016). All analyses were performed on an Agilent 1290 UHPLC system coupled to an Agilent 6470 triple-quadrupole mass spectrometer equipped with an electrospray ionization (ESI) source, without biological replication.

Fresh mycelia (100 mg) or sclerotia (50 mg) were accurately weighed into 5 mL centrifuge tubes. One milliliter of 50% acetonitrile was added, and the samples were homogenized thoroughly for 5 min. The mixtures were vortex-mixed and centrifuged at 12,000 rpm for 10 min at room temperature. The resulting supernatants were filtered through 0.22 μm membrane filters and transferred to LC vials for instrumental analysis.

Chromatographic separation was performed on a Waters BEH Amide column (2.1 × 100 mm, 1.7 μm) maintained at 35 °C. The mobile phases consisted of solvent A, water containing 0.15% formic acid and 10 mM ammonium formate (Sinopharm Chemical Reagent Co., Ltd., Shanghai, China), and solvent B, 100% acetonitrile (ACS reagent grade) containing 0.15% formic acid. The flow rate was set at 0.35 mL/min, and the injection volume was 2.0 μL. The gradient elution program was as follows (Table 1).

**Table 1.** Gradient elution program for HILIC–LC–MS/MS analysis.

**Time (min)**	**Flow rate (mL/min)**	**A%**	**B%**
0	0.35	0.0	100.0
6	0.35	0.0	100.0
6.1	0.35	5.9	94.1
10	0.35	17.6	82.4
12	0.35	29.4	70.6
12.5	0.35	29.4	70.6
12.8	0.35	0.0	100.0
15	0.35	0.0	100.0

The mass spectrometer was operated in positive electrospray ionization mode (ESI^+^) and multiple reaction monitoring (MRM) mode. The monitored transitions were *m/z* 148 → 84 for glutamate and *m/z* 104 → 87 for GABA. The cone voltage and collision energy were set at 10 V and 20 eV, respectively. External calibration curves were constructed using standard solutions of glutamate and GABA (Shanghai Yuanye Bio-Technology Co., Ltd., Shanghai, China). Good linearity was obtained (*r^2^* > 0.999). Quantification was performed using external standard calibration without internal standard correction.

The final concentrations of glutamate and GABA in the samples were expressed as mg/kg (fresh weight basis):

$W\left(\frac{\mathrm{mg}}{\mathrm{kg}}\right)=\frac{\left(C-C_{0}\right)\times V\times N}{m\times 1000}$,

where:

*W* is the analyte content in the sample (mg/kg);

*C* is the analyte concentration in the filtrate (µg/L);

*C*_0_ is the blank concentration (µg/L);

*V* is the final volume of extract (mL);

*N* is the dilution factor;

and *m* is the sample mass (g).

### Data analysis and visualization

All bar charts were generated using GraphPad Prism version 9.0 software (Dotmatics, Boston, MA, USA). Radial growth data of *Xylaria* sp. strain Mg-M4 on different media were analyzed using IBM SPSS Statistics for Windows version 29.0 (IBM Corp., Armonk, NY, USA). Prior to analysis, data were tested for normality and homogeneity of variances; one-way analysis of variance (ANOVA) was applied when these assumptions were met (*P* > 0.05). When significant differences were detected and multiple groups were compared, Tukey’s honestly significant difference (HSD) post hoc test was used.

## Results

### Induction and isolation of sclerotium-forming *Xylaria*

**Figure 2.**
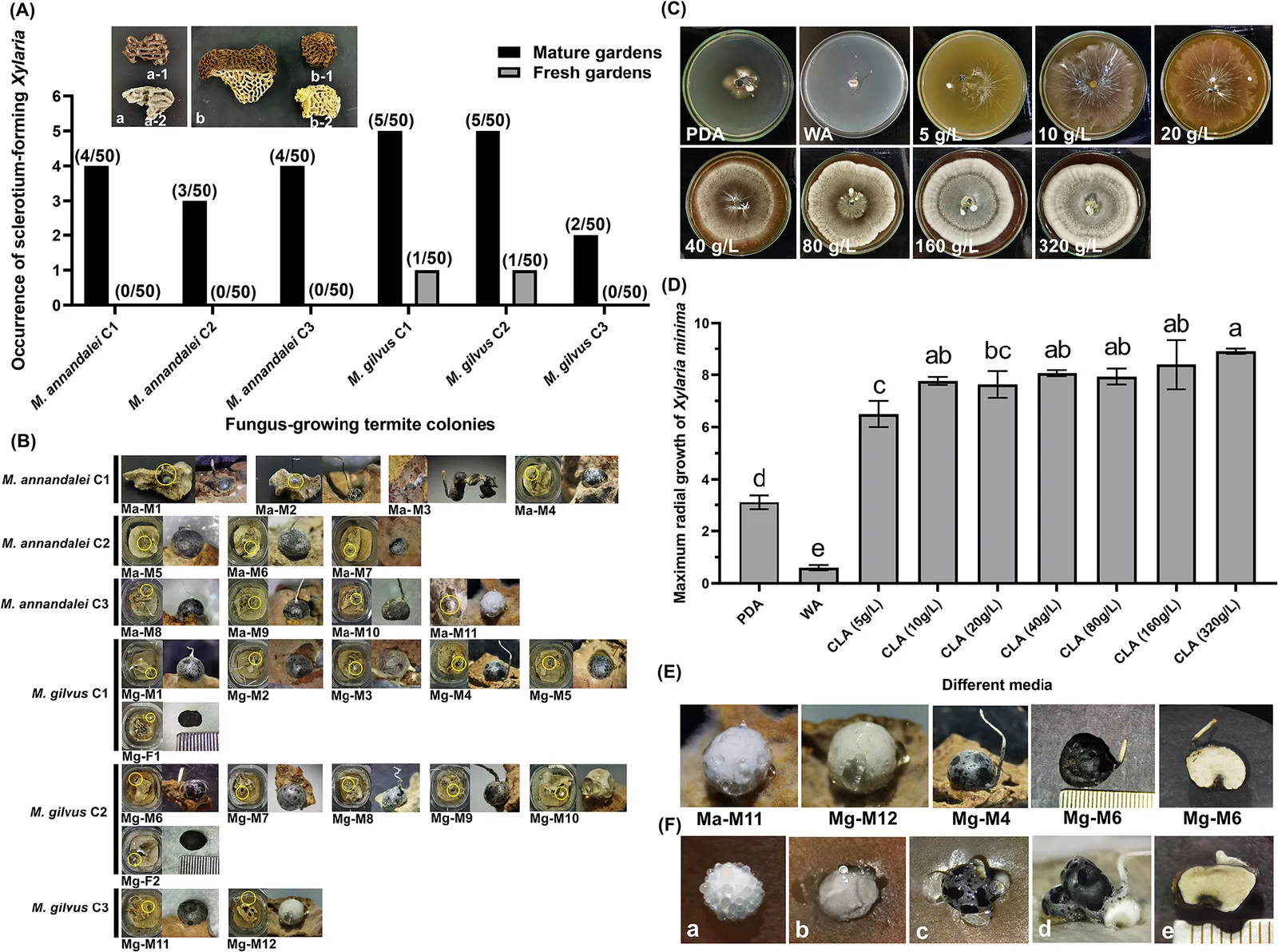
Induction of sclerotia and radial growth of *Xylaria* sp. strain Mg-M4 on different culture media. **A, B**. Induction rates of sclerotia from the termite fungus combs of *M. annandalei* and *M. gilvus* after 15 days of incubation. Fresh comb (a-1) and mature combs (a-2) of *M. annandalei* and fresh comb (b-1) and mature combs (b-2) of *M. gilvus*; **C, D**. Sclerotium-forming *Xylaria* sp. strain Mg-M4 growing on PDA, WA, and different concentrations of termite-comb leachate agar and its radial growth rates on the corresponding media on day 15. Bars sharing the same letter were not significantly different according to Tukey’s HSD post hoc test; **E**. Sclerotia forming and developing on termite fungus combs, with a vertical sectional view; **F**. Sclerotia forming and developing on 40 g/L termite-comb leachate agar, with a vertical sectional view.

Three hundred fresh fungus comb fragments and 300 mature comb fragments were collected from colonies of *M. annandalei* (C1–C3) and *M. gilvus* (C1–C3). In total, 300 comb pieces of *M. annandalei* were incubated, and only 11 boxes containing mature fungus combs developed sclerotia after 15 days of incubation; no sclerotia were observed on incubated fresh fungus combs. In 2 g mature fungus combs of *M. annandalei*, the induction rate of sclerotia was 7.3% (Fig. 2A). Similarly, among 300 incubated comb pieces from *M. gilvus* colonies (C1–C3), 12 boxes containing mature fungus combs formed sclerotia after 15 days. Interestingly, nearby fresh combs also formed two sclerotia, although the sclerotia were not attached to the fungus combs (Fig. 2B: Mg-F1, F2). In 2 g mature fungus combs of *M. gilvus*, the induction rate of sclerotia was 8%, whereas in 2 g fresh fungus gardens, the rate was only 1.3%. In the induction assay, sclerotia were primarily formed on mature fungus combs, except in two cases in which sclerotia formed on fresh combs but were not directly attached to the comb material. In total, 25 sclerotia were observed across all incubated fungus combs, but only 12 were successfully isolated and cultured.

### Morphological and phylogenetic identification

**Figure 3.**
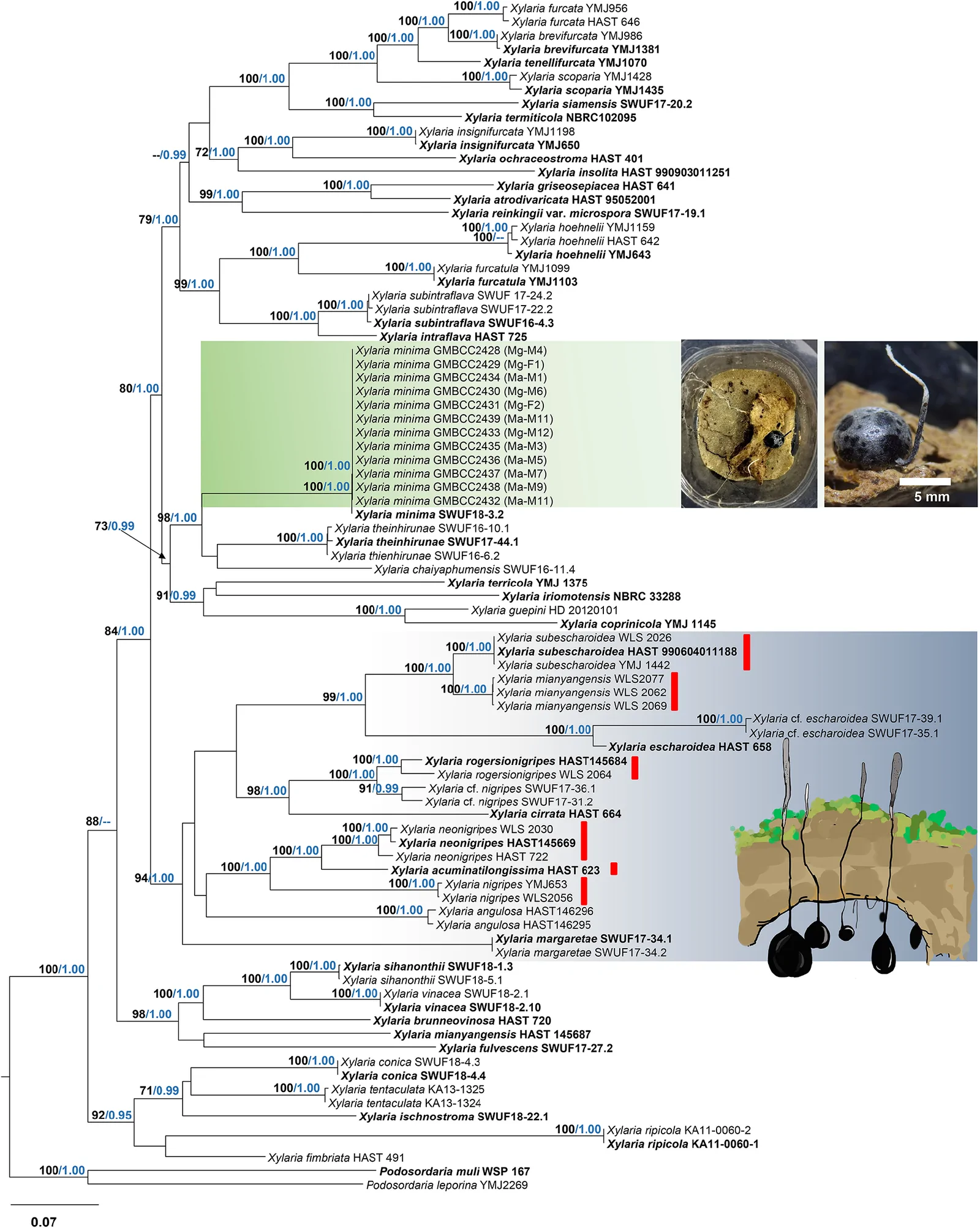
The *Xylaria* (TE clade) phylogram constructed based on combined ITS, *ACT*, *TUB2*, and *RPB2* sequences. Type strains are in bold. Phylogenetic analysis reveals that the small- and massive-sclerotium-forming species are distributed in distinct subclades. Specifically, the newly generated small-sclerotium-forming strains belonging to *X. minima* are highlighted in pale green, while the massive-sclerotium-forming *X. nigripes* species complex is highlighted in blue. Within this blue shading, the species-specific clades of the six previously reported massive-sclerotium-forming species are indicated by red blocks adjacent to the terminal branches.

Sclerotia matured from white to gray to dark black; as they matured, the outer hyphae gradually formed a very hard shell, either smooth or bumpy, with only slight increases in size over time and often with water droplets observed on the surface (Fig. 2E, F). Microscopic examination revealed that the sclerotia consisted solely of hyphae. After 15 days, the length (5.24 ± 1.68 mm), height (4.22 ± 1.34 mm), and wet weight (61.65 ± 37.21 mg) were measured and expressed as mean ± SD (*n* = 25) (Suppl. material 2: table SS3). After approximately 1 month, sclerotized hyphae broke through the sclerotial surface and extended upward, sometimes with conidia forming around their tips (Fig. 2, Suppl. material 1: fig. S1). The microscopic characteristics of the strain were observed on PDA after 1 month. Anamorph: Conidiogenous cells 1.5–2 × 1–2 μm (x– = 1 × 1.5 μm, *n* = 20), tiny, inconspicuous, oblong, and located terminally and laterally or at the apices of conidiophores. Conidia 2.5–3.5 × 1.5–2.5 μm (x– = 3 × 2 μm, *n* = 20), solitary or catenate, obovoid, hyaline, aseptate, and smooth-walled. The anamorph observed in the isolates was similar to that of the holotype of *X. minima* (SWUF18-3.2) described by Wangsawat et al. (2021) (Suppl. material 1: fig. S1). The multilocus phylogenetic analysis supported the placement of the isolates within *X. minima* (Fig. 3). However, the teleomorph was not observed in this study.

**Figure 4.**
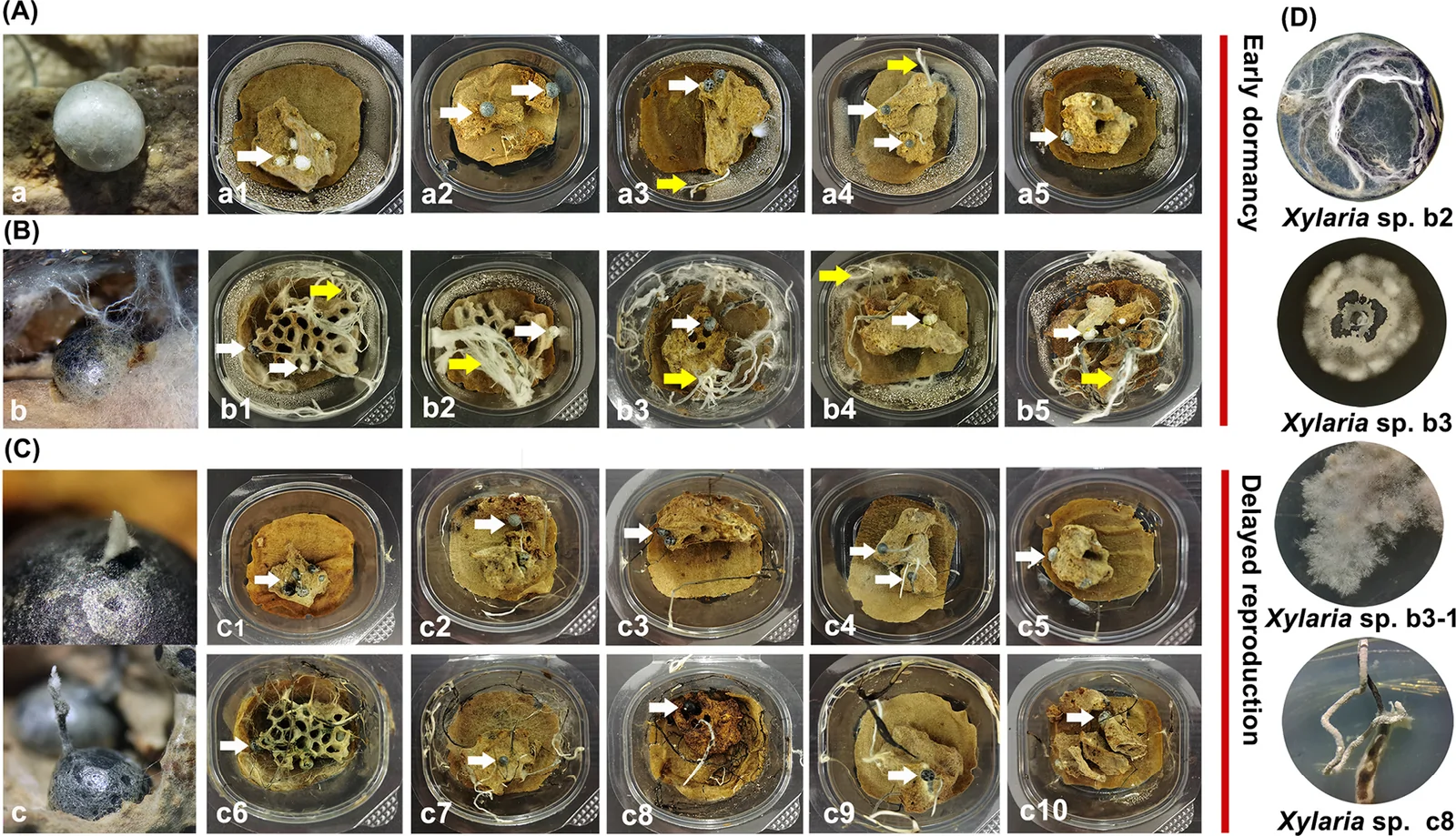
Growth of *X. minima* and other comb-specialized *Xylaria* species. **A**. *Xylaria minima* rapidly formed small sclerotia without exhibiting distinct mycelial growth during the first 10 days of incubation (a1–a5); **B**. *Xylaria minima* formed small sclerotia, accompanied by extensive mycelial growth of another comb-specialized *Xylaria* species during the same period (b1–b5); **C**. Later, *X. minima* mycelia emerged from the sclerotial surface and occasionally extended upward to produce conidia, whereas some mycelial death of the other *Xylaria* was observed (c1–c10); **D**. The isolated *Xylaria* strains from boxes growing on PDA, with strain names indicating the corresponding box numbers. Note: The white arrow indicates sclerotia of *X. minima*, and the yellow arrows indicate the mycelia of the competing *Xylaria* species.

The concatenated ITS, *ACT*, *TUB2*, and *RPB2* sequence dataset comprised 87 strains, with *Podosordaria leporina* (YMJ2269) and *P. muli* (WSP 167) as the outgroup taxa (Fig. 4). The concatenated sequence matrix comprised 4,390 characters, including gaps (ITS: 1–730 bp; *ACT*: 733–1,124 bp; *TUB2*: 1,127–3,160 bp; *RPB2*: 3,164–4,390 bp). A phylogenetic investigation based on ML analysis was conducted using the best RAxML tree, with a final likelihood value of −62,896.671141 (Fig. 4). The matrix contained 2,447 distinct alignment patterns, with 31.48% of characters undetermined (gaps). Estimated base frequencies were as follows: A = 0.227716, C = 0.285817, G = 0.241220, and T = 0.245247, with substitution rates AC = 1.286503, AG = 4.207724, AT = 1.146451, CG = 1.120540, CT = 6.519108, and GT = 1.000000; gamma distribution shape parameter α = 1.191341; tree length = 7.620992. The final average standard deviation of split frequencies at the end of the total MCMC generations was 0.009748 in the BI analysis. The 12 isolates (GMBCC2428, GMBCC2429, GMBCC2430, GMBCC243, GMBCC2432, GMBCC2433, GMBCC2434, GMBCC2435, GMBCC2436, GMBCC2437, GMBCC2438, and GMBCC2439) grouped with *X. minima* (SWUF18-3.2, type) with 100% ML/1.00 BYPP statistical support. *Xylaria minima* (SWUF18-3.2, type) was isolated from termite nests and produced cylindrical, unbranched stromata (Wangsawat et al. 2021), whereas the *X. minima* isolates in this study occurred in the form of sclerotia. Although *X. minima* was identified as an independent clade (highlighted in pale green), it was phylogenetically distant from the massive sclerotium-forming clade (see blue clade in Fig. 3).

### Timing of the production of small sclerotia

Sclerotia varied in shape from spherical to ovoid or irregular (Fig. 2B). Some sclerotia formed rapidly on incubated 2 g fungus combs within a few days, whereas others required a longer period. However, almost all observed sclerotia were produced during the 10-day incubation period (Fig. 4A, B). Sclerotia formed without undergoing a typical vegetative growth phase or developing sclerotized hyphae (mycelial cords) (Figs 2E, 4A, 4B). Instead, during the initial stage, small, spherical, white sclerotia suddenly appeared on the surface of the fungus garden, as well as beneath and within its galleries (Suppl. material 1: fig. S2). Small water droplets were sometimes visible on their surface; these droplets likely represented guttation exudates produced during periods of rapid nutrient accumulation. In the boxes where sclerotia formed, some were accompanied by visible, extensive mycelial growth of other comb-specialized *Xylaria* species (Fig. 4B: b1–b5). ITS BLASTn results indicated that the potential nutrient competitors within the nest were not *X. minima* (Suppl. material 2: table S4). After approximately 20–30 days at room temperature, almost simultaneously with the decline of vegetative hyphal growth in other *Xylaria*, an unbranched germinating hypha broke through the *X. minima* sclerotial husk and extended upward for conidial release (Figs 4C: c1–c10, Suppl. material 1: fig. S1).

### The formation of sclerotia is not restricted to termite fungus combs

After *Xylaria* sclerotium (Mg-M4) was isolated and grown on PDA for 1 month, 0.5 cm mycelial plugs were transferred to agar media containing various concentrations of termite-comb leachate, PDA, and WA. Radial growth differed significantly among media (one-way ANOVA: *F* [8, 18] = 129.6, *P* < 0.0001); Tukey’s HSD post hoc tests indicated that the radial growth of Mg-M4 did not differ significantly among the different concentrations of termite-comb leachate media on day 15 but was significantly faster than that on PDA and WA (Fig. 2C, D). The extent of mycelial biomass differed markedly among treatments, with visibly greater growth at higher concentrations (80 g/L, 160 g/L, and 320 g/L) (Fig. 2C, Suppl. material 1: fig. S3). Sclerotia were produced at concentrations of 5, 10, 20, and 40 g/L (w/v) within 15 days; in contrast, sclerotia rarely formed on PDA or WA. Sclerotia formed on CLA varied in shape from spherical to ovoid or irregular and were produced in a manner similar to those formed on incubated fungus combs (Fig. 2E, F). Termite-comb leachate agar (CLA) increased sclerotial formation and mycelial growth in *X. minima*.

**Figure 5.**
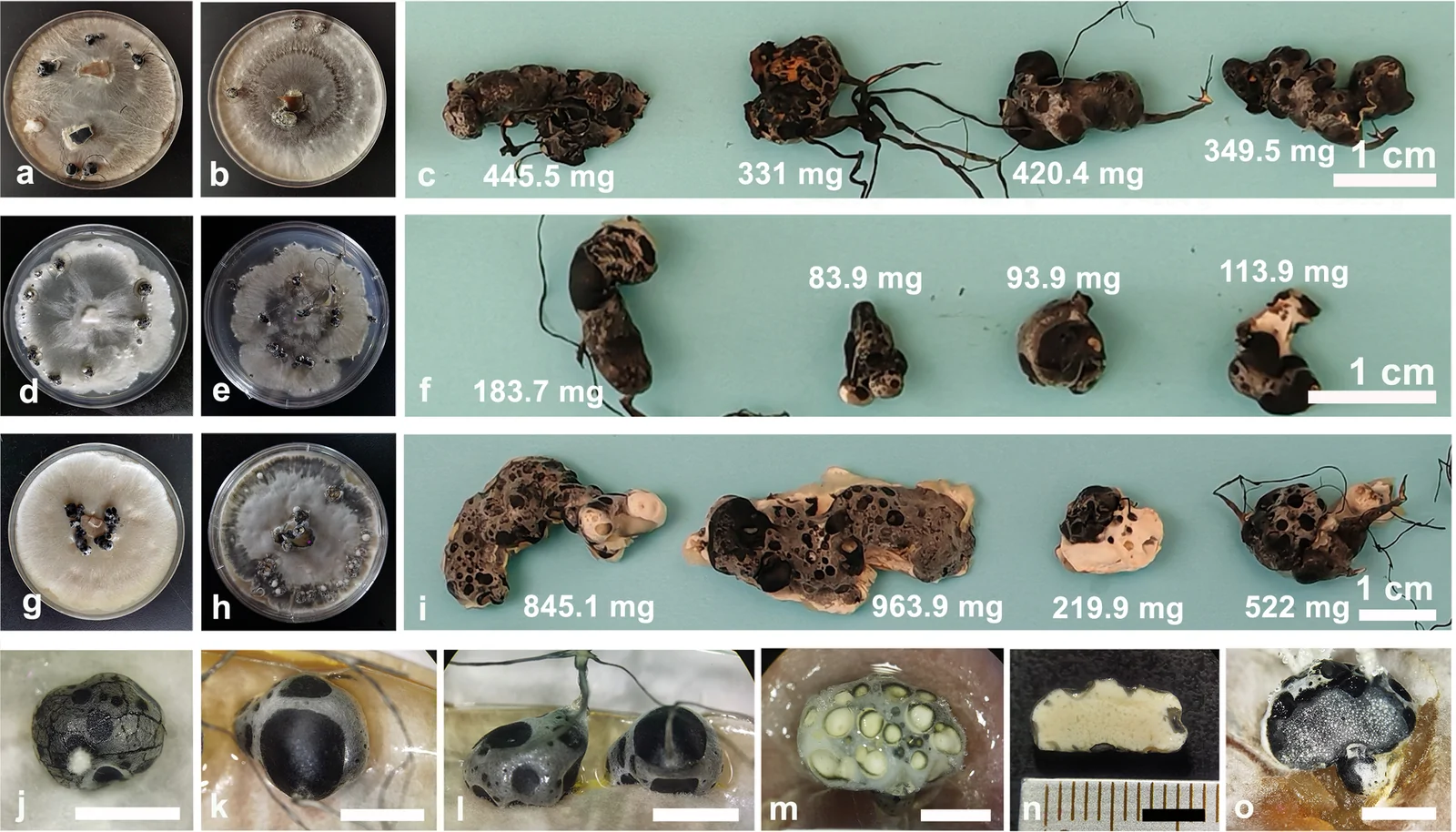
Representative examples of the sclerotia produced on media. **a–c**. Sclerotia forming on comb leachate medium (40 g/L) after 1 month; **d–f**. Sclerotia forming on PDA after 2 months; **g–i**. Sclerotia forming on oat medium after 2 months; **c, f, i**. Harvested sclerotia from PDA, CLA, and oat media after 2 months; **j**. Formation of sclerotia on PDA; **k–o**. Formation of sclerotia on CLA and their vertical sections. Scale bar: 5 mm (**j–n**).

Strains isolated from sclerotial tissues rarely produced sclerotia when cultured on PDA; instead, they grew slowly and formed densely aggregated hyphae (mycelial cords). However, after repeated subculturing on CLA, the *Xylaria* isolates occasionally produced sclerotia more readily on PDA and oat media. Representative plates are shown in Fig. 5. *Xylaria minima* grew well and formed sclerotia on termite-comb leachate agar (40 g/L) prepared from the termite fungus combs of *Macrotermes* (Fig. 2C). After 2–3 months, densely aggregated hyphae were generated mainly from the upper surface of mature sclerotia, and the sclerotia gradually became hollow after 3 months (Fig. 5). The formation of sclerotia on CLA plates and their vertical sections are presented in Fig. 5j–o. After sectioning, the sclerotial tissues appeared flesh-colored; however, both the wounded surfaces and sclerotial surfaces rapidly blackened and hardened within a few days after exposure to the atmosphere. Therefore, *X. minima* appears to be readily domesticated and shows no strict substrate specificity. The CLA transition medium significantly promoted rapid sclerotium formation and mycelial growth.

### The possibility of substrate cultivation of *Xylaria minima*

**Figure 6.**
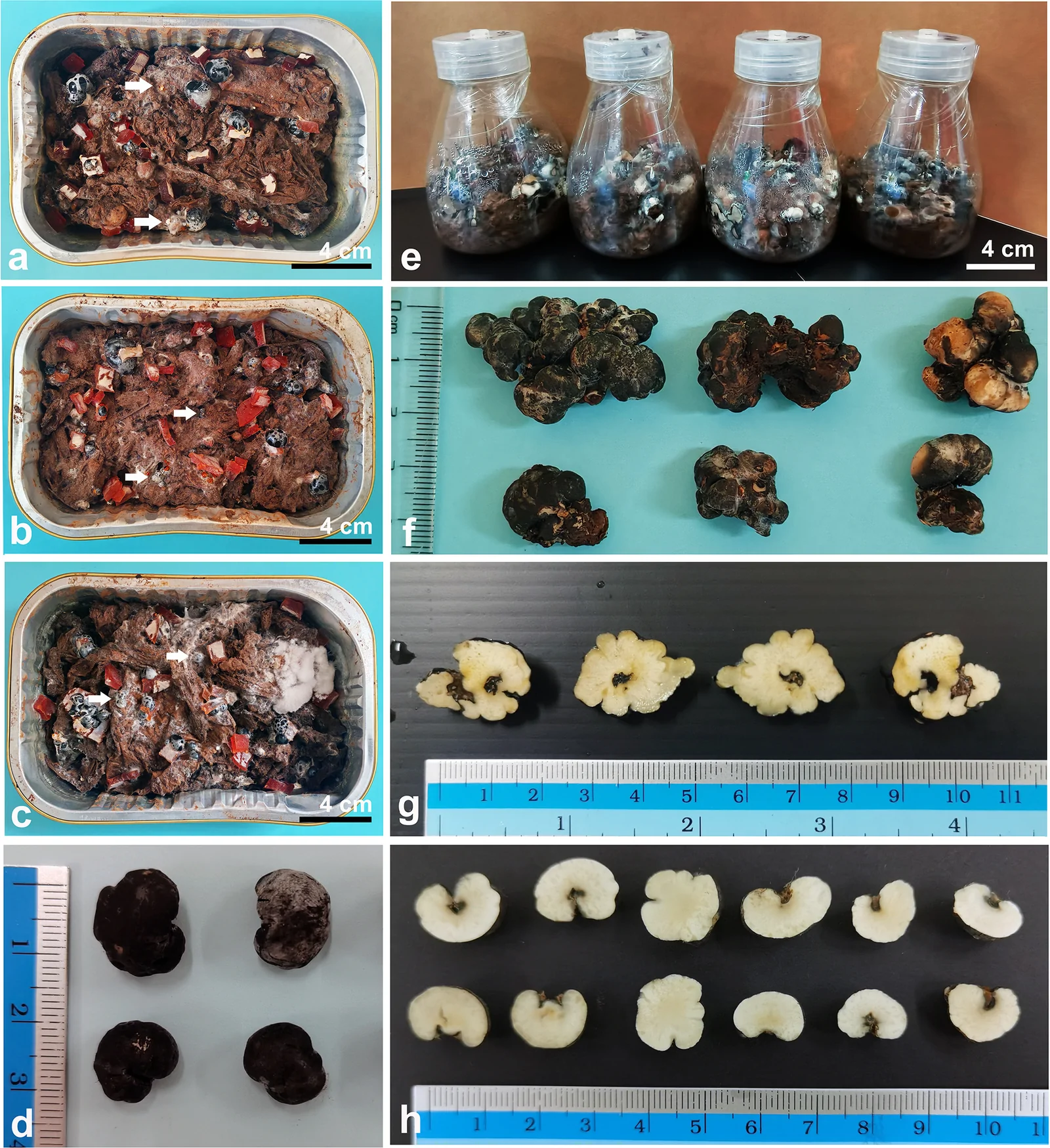
Sclerotia formed on comb-substitute substrates. **a–c**. Sclerotia forming on paper-based soil substrates autoclaved three times (1 month); the white arrow indicates newly formed sclerotia on the soil; **d, h**. Examples of harvested large sclerotia from boxes containing soil and their sections; **e**. Sclerotia forming on agricultural substrates autoclaved three times in a sterilized flask (1 month); **f, g**. Examples of harvested large sclerotia from a flask containing agricultural substrates and their sections.

Mycelia grown on PDA and CLA were used as inocula for substrate cultivation in Erlenmeyer flasks containing repeatedly sterilized mature fungus combs, paper sheets, and water. Within 1 month, large sclerotia of *X. minima* developed. These sclerotia formed through the aggregation of numerous smaller sclerotia into compact clusters (Suppl. material 1: fig. S4), with the largest individual compact sclerotium reaching 4.262 g (Table 2). When paper-based agricultural substrates (PAS) were used to replace the comb substrate, *X. minima* strain Mg-M4 also grew well and readily produced large sclerotia in Erlenmeyer flasks (Fig. 6e, f). Extensive vegetative mycelial growth was not observed, and the sclerotia produced in the substrate could potentially be harvested repeatedly. The maximum sclerotium weights produced on the comb material and agricultural substrate were comparable (4.262 vs. 3.808 g) (Table 2). Moreover, in square boxes, when paper-based soil substrate (PSS) was used as the only substrate, the maximum sclerotium weight was similar to that obtained on PDA and oat media (approximately 1 g) (Figs 5, 6).

**Table 2.** Sclerotium formation of *X. minima* on different media and substrates after 1 month.

**Inoculum source**	**Medium/substrate**	**Sclerotium formation**	**Maximum sclerotium weight (g)**
Original sclerotial tissue	PDA	Absent	Rarely observed
	WA	Absent	Rarely observed
CLA-grown mycelia	PDA	Present	0.184
	Oat media	Present	0.964
	Paper-based comb substrates (PCS)	Present	4.262
	Paper-based soil substrates (PSS)	Present	1.276
PDA-grown mycelia	Paper-based agricultural substrate (PAS)	Present	3.808

### Chemical constituents detected in PDA-grown mycelia and PAS-formed sclerotia

From the preliminary analysis of chemical constituents in sclerotial and mycelial samples, sclerotia consistently exhibited higher levels of all major classes of metabolites than mycelia (Table 3). On a per-100 g basis, crude polysaccharides, total alkaloids, total sterols, and total triterpenoids were elevated by approximately 1.5–2-fold in sclerotia. The specific metabolites γ-aminobutyric acid (GABA) and L-glutamic acid were also markedly enriched, with 3- and 6.7-fold increases, respectively. Due to the absence of biological replicates, data are presented as single values and are intended only for preliminary comparison; no statistical analysis was performed.

**Table 3.** Quantitative comparison of major classes of chemical constituents, γ-aminobutyric acid (GABA), and L-glutamic acid in fresh mycelia and fresh sclerotia, including fold increases in sclerotia.

**Class**	**Tested compounds**	**Mycelia**	**Sclerotia**	**Fold increase**
Major class	Crude Polysaccharides	0.240 g/100 g	0.44 g/100 g	1.83-fold
	Total Alkaloids	0.01 g/100 g	0.02 g/100 g	2.00-fold
	Total sterols	0.240 g/100 g	0.372 g/100 g	1.55-fold
	Total Triterpenoids	0.0362 g/100 g	0.0757 g/100 g	2.09-fold
Specific compounds	L-glutamic acid	85.09 mg/kg	576.51 mg/kg	6.77-fold
	γ-Aminobutyric acid (GABA)	3.73 mg/kg	11.90 mg/kg	3.19-fold

## Discussion

Phylogenetic analysis places the small sclerotium-forming *X. minima* in an independent subclade, separate from the previously recognized massive sclerotium-forming group. This contrasts with the suggestion by Hsieh and Ju (2024) that the sclerotium-forming habit arose only once within termite-associated *Xylaria*. Instead, the results indicate that the sclerotium-forming habit in comb-specialized *Xylaria* has evolved at least twice, or possibly multiple times. Ecologically, *X. minima* in Xishuangbanna, China, was induced almost exclusively from mature fungus combs of *Macrotermes* spp., with only occasional records from fresh combs. However, these findings are based on only two *Macrotermes* species from a single geographic locality, and broader sampling is needed.

Multiple substrates were evaluated to assess the feasibility of cultivating *X. minima* for sclerotium production under laboratory conditions. CLA was used to domesticate the strain toward faster growth and enhanced sclerotium formation. Although the underlying mechanism remains unclear, the observed enhancement may result from the nutrient-rich CLA or from other unknown factors that upregulate genes related to sclerotium formation. When comb material and agricultural substrates were used to cultivate *X. minima* sclerotia, the fungus produced large aggregates of numerous smaller sclerotia, with individual sclerotia reaching small truffle-like dimensions (Cseh et al. 2024). Notably, extensive vegetative mycelial growth was not observed in these substrates (Figs 2, 4), suggesting high nutrient-use efficiency and effective conversion of the sparse mycelium into sclerotial biomass. Sclerotia formed rapidly within 1 month and could be harvested repeatedly. Further optimization of substrates and cultivation strategies is necessary to unlock the agricultural and commercial potential of *Xylaria* sclerotia.

Sclerotia exhibited a pronounced capacity to enrich chemical constituents compared with mycelia. Total alkaloids and total triterpenoids in sclerotia were elevated approximately twofold, and the specific metabolites γ-aminobutyric acid (GABA) and L-glutamic acid were also markedly enriched, showing 3- and 6.7-fold increases, respectively. Previous studies have detected GABA and glutamic acid in Wuling capsule™, a preparation derived from *X. nigripes* (Chen et al. 1990; He and Liu 2010), and the commercial product GABA-rich Zylaria™, containing mycelial extracts of *X. nigripes*, has been reported to promote relaxation and improve sleep quality through its natural tranquilizing properties (Deshmukh et al. 2021). These findings further highlight the value of sclerotium cultivation. However, the results are limited by the inconsistency in the substrates used; the lower chemical content of mycelia grown on PDA compared with sclerotia produced on PAS may reflect the fact that agricultural substrates provide a more natural, chemically complex environment, potentially favoring a broader range of secondary metabolites in sclerotia. This is consistent with previous reports that PDA significantly alters fungal secondary-metabolite profiles, highlighting its limitations for metabolite-based research (Westphal et al. 2021). Therefore, the selection of appropriate, uniform cultivation substrates is essential for chemical comparisons between sclerotia and mycelia.

Another limitation of this study is that, for the newly identified sclerotium-forming species *X. minima*, preliminary chemical measurements were performed on fresh samples without biological replicates; chemical concentrations are expected to be higher on a dry-weight basis, making direct and precise comparisons with previous findings difficult. For example, Chen et al. (2014) reported that, under optimized shake-flask conditions, *X. nigripes* produced extracellular polysaccharides at 2.36 ± 0.18 mg/mL in the broth, along with intracellular polysaccharides and triterpenoids at 2.38 ± 0.07 mg/g and 31.29 ± 1.17 mg/g of dry mycelial weight, respectively. Liang et al. (2025) further emphasized that polysaccharides constitute a major component of the chemical composition of *X. nigripes*, particularly in crude form. In addition, Liang et al. (2025) highlighted the chemical diversity of 82 compounds identified from *X. nigripes* between 2004 and October 2024, encompassing alkaloids, sterols, chromogenic ketones, terpenoids, isocoumarin derivatives, resorcinol derivatives, and naphthalenone derivatives, among which 26 compounds exhibit diverse pharmacological properties. Therefore, future studies should evaluate specific chemical compounds and their bioactivities on a dry-weight basis to facilitate direct comparison with data reported for other medicinal and edible fungi, particularly the sclerotia of the commercial medicinal fungus *X. nigripes*.

On incubated termite fungus combs, *X. minima* rapidly forms small, spherical sclerotia within the first few days, bypassing a distinct vegetative growth phase (this study). Such accelerated development often allows *X. minima* to emerge even faster than other fungal weeds (Fig. 4A: a1–a5; Suppl. material 2: table S4). During the period of weed expansion, such rapid and inconspicuous sclerotium formation may help *X. minima* conceal itself and quickly occupy small ecological niches (Fig. 4B: b1–b5). As no distinct mycelial emergence was observed on the comb surface, the vegetative phase may have occurred within the comb interior. Meanwhile, sclerotia were observed forming on the comb surface, beneath the sides, and within the galleries of the incubated combs, which together may allow *Xylaria* to evade termite hygienic behaviors (Fig. 4, Suppl. material 1: fig. S2). In addition, sclerotia of *X. minima* may function as durable, dormant propagules, which likely increase their resistance to continuous mechanical removal by termites, termites’ antimicrobial secretions, and fungistatic microbes and other fungal weeds. When termites later disappear and the expansion of weedy fungi slows, the sclerotia typically germinate by producing thin, elongated mycelial cords (Fig. 4C: c1–c10). Together, rapid sclerotial formation, dormancy, and temporally delayed emergence are hypothesized to constitute an adaptive strategy that enables *Xylaria* to exploit the termite–fungus symbiosis. To validate this hypothesis, future research should investigate a wider diversity of small sclerotium-forming *Xylaria* species and their termite hosts, with particular emphasis on termite behavioral responses to sclerotia and on the profiles of volatile organic compounds (VOCs) emitted during the mycelial and sclerotial stages.

## Additional information

### Artificial Intelligence (AI) use

The authors accept full responsibility for the content of the manuscript, including the disclosure of any use of AI.

No AI tools were used in the preparation of this manuscript.

### Author ORCIDs

E.-F. Yang https://orcid.org/0000-0003-2385-6402

M. Stadler https://orcid.org/0000-0002-7284-8671

A. Möllerke https://orcid.org/0009-0003-3944-520X

M.C. Samarakoon https://orcid.org/0000-0002-4815-125X

C.-I. Chiu https://orcid.org/0000-0002-1603-0736

D.-Q. Dai https://orcid.org/0000-0001-8935-8807

I. Promputtha https://orcid.org/0000-0003-3376-4376

F.-Q. Yu https://orcid.org/0000-0001-7931-080X

S. Ali https://orcid.org/0000-0002-3230-1436

S.C. Karunarathna https://orcid.org/0000-0001-7080-0781

S. Tibpromma https://orcid.org/0000-0002-4706-6547

J.-Y. Yang https://orcid.org/0009-0000-0375-990X

### Data availability

All of the data that support the findings of this study are available in the main text or Supplementary Information.

## Supplementary materials

Supplementary material 1 Supplementary figures Data type docx https://binary.pensoft.net/file/1751702This dataset is made available under the Open Database License (http://opendatacommons.org/licenses/odbl/1.0/). The Open Database License (ODbL) is a license agreement intended to allow users to freely share, modify, and use this Dataset while maintaining this same freedom for others, provided that the original source and author(s) are credited. Er-Fu Yang, Marc Stadler, Anton Möllerke, Milan C. Samarakoon, Chun-I Chiu, Dong-Qin Dai, Itthayakorn Promputtha, Fu-Qiang Yu, Sajad Ali, Samantha C. Karunarathna, Saowaluck Tibpromma, Jing-Ya Yang

Supplementary material 2 Supplementary tables Data type docx https://binary.pensoft.net/file/1751703This dataset is made available under the Open Database License (http://opendatacommons.org/licenses/odbl/1.0/). The Open Database License (ODbL) is a license agreement intended to allow users to freely share, modify, and use this Dataset while maintaining this same freedom for others, provided that the original source and author(s) are credited. Er-Fu Yang, Marc Stadler, Anton Möllerke, Milan C. Samarakoon, Chun-I Chiu, Dong-Qin Dai, Itthayakorn Promputtha, Fu-Qiang Yu, Sajad Ali, Samantha C. Karunarathna, Saowaluck Tibpromma, Jing-Ya Yang

## References

[B1] Agarwal R, Gupta M, Sen R, Panchal A, ES N, Raychoudhury R (2024) Investigation into how *Odontotermes obesus* maintains a predominantly *Termitomyces* monoculture in their fungus combs suggests a potential partnership with both fungi and bacteria. Communications Biology 7(1): 1010. 10.1038/s42003-024-06708-2 PMC11330501 39154098

[B2] Barakat H, Aljutaily T (2025) Role of γ-Aminobutyric acid (GABA) as an inhibitory neurotransmitter in diabetes management: mechanisms and therapeutic implications. Biomolecules 15(3): 399. 10.3390/biom15030399 PMC11940341 40149935

[B3] Batra LR, Batra SWT (1979) Termite-fungus mutualism. In: Batra LR (Ed.) Insect-fungus Symbiosis: Mutualism and Commensalism. Allanheld, Osmun & Co., Montclair, New Jersey, 117–163.

[B4] Buck K, Voehringer P, Ferger B (2009) Rapid analysis of GABA and glutamate in microdialysis samples using high performance liquid chromatography and tandem mass spectrometry. Journal of Neuroscience Methods 182(1): 78–84. 10.1016/j.jneumeth.2009.05.018 19505500

[B5] Carbone I, Kohn LM (1999) A method for designing primer sets for speciation studies in filamentous ascomycetes. Mycologia 91(3): 553–556. 10.1080/00275514.1999.12061051

[B6] Chang CK, Ho WJ, Chang SL, Yeh CH, Liang ZC, Hsu TH, Hsieh CW (2018) Fractionation, characterization and antioxidant activity of exopolysaccharide from fermentation broth of a *Xylaria nigripes*. Bioactive Carbohydrates and Dietary Fibre 16: 37–42. 10.1016/j.bcdf.2018.02.005

[B7] Chen JZ, Lo HC, Lin FY, Chang SL, Hsieh C, Liang ZC, Ho WJ, Hsu TH (2014) Effects of medium components and culture conditions on mycelial biomass and the production of bioactive ingredients in submerged culture of *Xylaria nigripes* (*Ascomycetes*), a Chinese medicinal fungus. International Journal of Medicinal Mushrooms 16(5): 431–447. 10.1615/IntJMedMushrooms.v16.i5.30 25271979

[B8] Chen WR, Fang HF, Ma ZZ, Ding RR (1990) Chemical constituents of *Xylaria nigripes*. Chinese Pharmaceutical Journal 25: 647–649. [in Chinese]

[B9] Cseh P, Merényi Z, Bóna L, Varga T, Bóka K, Nagy I, Kaounas V, Vidal JM, Paz A, Bratek Z (2024) Taxonomic characterisation of the *Regianum* clade (genus *Tuber*) and the trait evolution of spore size among true truffles. Mycological Progress 23(1): 11. 10.1007/s11557-024-01949-1

[B10] Deshmukh SK, Sridhar KR, Gupta MK (2021) Application of selected species of the genus *Xylaria* in traditional medicine. In: Deshmukh SK, Sridhar KR (Eds) Advances in Macrofungi: Pharmaceuticals and Cosmeceuticals. CRC Press, Boca Raton, FL, USA, 122–136. 10.1201/9781003191278-11

[B11] Divate RD, Chung YC (2017) *In vitro* and *in vivo* assessment of anti-inflammatory and immunomodulatory activities of *Xylaria nigripes* mycelium. Journal of Functional Foods 35: 81–89. 10.1016/j.jff.2017.05.027

[B12] DuBois M, Gilles KA, Hamilton JK, Rebers PA, Smith F (1956) Colorimetric method for determination of sugars and related substances. Analytical Chemistry 28(3): 350–356. 10.1021/ac60111a017

[B13] Eckstein JA, Ammerman GM, Reveles JM, Ackermann BL (2008) Analysis of glutamine, glutamate, pyroglutamate, and GABA in cerebrospinal fluid using ion pairing HPLC with positive electrospray LC/MS/MS. Journal of Neuroscience Methods 171(2): 190–196. 10.1016/j.jneumeth.2008.02.019 18433876

[B14] Erixon P, Svennblad B, Britton T, Oxelman B (2003) Reliability of Bayesian posterior probabilities and bootstrap frequencies in phylogenetics. Systematic Biology 52(5): 665–673. 10.1080/10635150390235485 14530133

[B15] Fricke J, Schalk F, Kreuzenbeck NB, Seibel E, Hoffmann J, Dittmann G, Conlon BH, Guo H, De Beer ZW, Vassão DG, Gleixner G, Poulsen M, Beemelmanns C (2023) Adaptations of *Pseudoxylaria* towards a comb-associated lifestyle in fungus-farming termite colonies. The ISME Journal 17(5): 733–747. 10.1038/s41396-023-01374-4 PMC10119272 36841903

[B16] Gan J, Feng Y, He Z, Li X, Zhang H (2017) Correlations between antioxidant activity and alkaloids and phenols of maca (*Lepidium meyenii*). Journal of Food Quality 2017(1): 3185945. 10.1155/2017/3185945

[B17] Glez-Peña D, Gómez-Blanco D, Reboiro-Jato M, Fdez-Riverola F, Posada D (2010) FALTER: Program oriented conversion of DNA and protein alignments. Nucleic Acids Research 38: 14–18. 10.1093/nar/gkq321 PMC2896128 20439312

[B18] Guedegbe HJ, Miambi E, Pando A, Roman J, Houngnandan P, Rouland-Lefevre C (2009) Occurrence of fungi in combs of fungus-growing termites (*Isoptera*: *Termitidae*, *Macrotermitinae*). Mycological Research 113(10): 1039–1045. 10.1016/j.mycres.2009.06.008 19576985

[B19] Haei M, Rousk J, Ilstedt U, Öquist M, Bååth E, Laudon H (2011) Effects of soil frost on growth, composition and respiration of the soil microbial decomposer community. Soil Biology and Biochemistry 43(10): 2069–2077. 10.1016/j.soilbio.2011.06.005

[B20] Hall TA (1999) BioEdit: a user-friendly biological sequence alignment editor and analysis program for Windows 95/98/NT. Nucleic Acids Symposium Series 41: 95–98.

[B21] He X, Liu P (2010) Determination of 14 kinds of amino acids in Wuling capsule by HPLC-FLD. Chinese Traditional Patent Medicine 32(8): 1358–1361. [in Chinese]

[B22] Hsieh HM, Ju YM (2024) *Xylaria* sclerotia formed within termite nests. In: Hsueh YP, Blackwell M (Eds) Fungal Associations. The Mycota; Springer, Cham, Switzerland, 333–356. 10.1007/978-3-031-41648-4_14

[B23] Hsieh HM, Lin CR, Fang MJ, Rogers JD, Fournier J, Lechat C, Ju YM (2010) Phylogenetic status of *Xylaria* subgenus *Pseudoxylaria* among taxa of the subfamily *Xylarioideae (Xylariaceae)* and phylogeny of the taxa involved in the subfamily. Molecular Phylogenetics and Evolution 54(3): 957–969. 10.1016/j.ympev.2009.12.015 20035889

[B24] Hsieh HM, Chou JC, Ju YM (2020) *Xylaria insolita* and *X. subescharoidea*: two newly described species collected from a termite nesting site in Hua-lien, Taiwan. Botanical Studies 61(1): 11. 10.1186/s40529-020-00287-1 PMC7136384 32253525

[B25] Inoue K, Miyazaki Y, Unno K, Min JZ, Todoroki K, Toyo’oka T (2016) Stable isotope dilution HILIC‐MS/MS method for accurate quantification of glutamic acid, glutamine, pyroglutamic acid, GABA and theanine in mouse brain tissues. Biomedical Chromatography 30(1): 55–61. 10.1002/bmc.3502 26033549

[B26] Jen CI, Su CH, Lu MK, Lai MN, Ng LT (2021) Synergistic anti‐inflammatory effects of different polysaccharide components from *Xylaria nigripes*. Journal of Food Biochemistry 45(4): e13694. 10.1111/jfbc.13694 33687093

[B27] Ju YM, Hsieh HM (2007) *Xylaria* species associated with nests of *Odontotermes formosanus* in Taiwan. Mycologia 99(6): 936–957. 10.1080/15572536.2007.11832525 18333517

[B28] Ju YM, Hsieh HM, He XS (2022) Wulingshen, the massive *Xylaria* sclerotia used as traditional Chinese medicine, is produced by multiple species. Mycologia 114: 175–189. 10.1080/00275514.2021.2005985 35073226

[B29] Katoh K, Standley DM (2013) MAFFT multiple sequence alignment software version 7: improvements in performance and usability. Molecular Biology and Evolution 30(4): 772–780. 10.1093/molbev/mst010 PMC3603318 23329690

[B30] Li G, Schmidt S, Silué SK, Koné NGA, Poulsen M (2025) Phylogenetic analysis of termite-associated *Xylaria* from Africa reveals hidden diversity. Fungal Biology 129(1): 101523. 10.1016/j.funbio.2024.12.001 39826975

[B31] Li J, Li LQ, Long HP, Liu J, Jiang YP, Xue Y, Wang WX, Tan GS, Gong ZC, Liu JK (2021) Xylarinaps A–E, five pairs of naphthalenone derivatives with neuroprotective activities from *Xylaria nigripes*. Phytochemistry 186: 112729. 10.1016/j.phytochem.2021.112729 33721798

[B32] Liang AL, Tan YF, Lu WY, Xu PJ, Yuan YY, Chen QA, Long HP, Wang WX, Li J (2025) A comprehensive review of the metabolites, biological activities, and clinical application of a medicinal fungus *Xylaria nigripes*. Journal of Ethnopharmacology 350: 120041. 10.1016/j.jep.2025.120041 40436127

[B33] Lin BB, Liu X, Wu SQ, Zheng HX, Huo KK, Qi SS, Chen C (2019) Phytochemicals content, antioxidant and antibacterial activities of *Sophora viciifolia*. Chemistry & Biodiversity 16(7): e1900080. 10.1002/cbdv.201900080 31111998

[B34] Lin Y, Wang XY, Ye R, Hu WH, Sun SC, Jiao HJ, Song XH, Yuan ZZ, Zheng YY, Zheng GQ, He JC (2013) Efficacy and safety of Wuling capsule, a single herbal formula, in Chinese subjects with insomnia: A multicenter, randomized, double-blind, placebo-controlled trial. Journal of Ethnopharmacology 145(1): 320–327. 10.1016/j.jep.2012.11.009 23178661

[B35] Liu YJ, Whelen S, Hall BD (1999) Phylogenetic relationships among ascomycetes: evidence from an RNA polymerase II subunit. Molecular Biology and Evolution 16(12): 1799–1808. 10.1093/oxfordjournals.molbev.a026092 10605121

[B36] Long HP, Zhou X, Zhou SQ, Li LQ, Liang AL, Lu WY, Wang WX, Liu S, Li J, Liu JK (2025) Five brasilane-type sesquiterpenoids with neuroprotective activities from *Xylaria nigripes*. Phytochemistry 231: 114357. 10.1016/j.phytochem.2024.114357 39662694

[B37] Miller MA, Pfeiffer W, Schwartz T (2010) Creating the CIPRES Science Gateway for inference of large phylogenetic trees. In Proceedings of the Gateway Computing Environments Workshop (GCE), 14 Nov. 2010. IEEE, New Orleans, LA, USA, 1–8. 10.1109/GCE.2010.5676129

[B38] Nylander JA, Wilgenbusch JC, Warren DL, Swofford DL (2008) AWTY (Are we there yet?): A system for graphical exploration of MCMC convergence in Bayesian phylogenetics. Bioinformatics 24(4): 581–583. 10.1093/bioinformatics/btm388 17766271

[B39] O’Donnell K, Cigelnik E (1997) Two divergent intragenomic rDNA ITS2 types within a monophyletic lineage of the fungus *Fusarium* are nonorthologous. Molecular Phylogenetics and Evolution 7(1): 103–116. 10.1006/mpev.1996.0376 9007025

[B40] Okane I, Hsieh HM, Ju YM, Lin CR, Huang CY, Kuan IC (2025) *Xylaria iriomotensis* sp. nov. from termite nests and notes on *X. angulosa*. Botanical Studies 66(1): 4. 10.1186/s40529-024-00447-7 PMC11748714 39820744

[B41] Pedrosa AM, de Castro WV, Castro AHF, Duarte-Almeida JM (2020) Validated spectrophotometric method for quantification of total triterpenes in plant matrices. DARU Journal of Pharmaceutical Sciences 28(1): 281–286. 10.1007/s40199-020-00342-z PMC7214563 32285314

[B42] Peng WF, Wang X, Hong Z, Zhu GX, Li BM, Li Z, Ding MP, Geng Z, Jin Z, Miao L, Wu LW, Zhan SK (2015) The anti-depression effect of *Xylaria nigripes* in patients with epilepsy: A multicenter randomized double-blind study. Seizure 29: 26–33. 10.1016/j.seizure.2015.03.014 26076841

[B43] Ronquist F, Teslenko M, Van Der Mark P, Ayres DL, Darling A, Höhna S, Larget B, Liu L, Suchard MA, Huelsenbeck JP (2012) MrBayes 3.2: efficient Bayesian phylogenetic inference and model choice across a large model space. Systematic Biology 61(3): 539–542. 10.1093/sysbio/sys029 PMC3329765 22357727

[B44] Sabir SM, Hayat I, Gardezi SDA (2003) Estimation of sterols in edible fats and oils. Pakistan Journal of Nutrition 2(3): 178–181. 10.3923/pjn.2003.178.181

[B45] Stamatakis A (2014) RAxML version 8: a tool for phylogenetic analysis and post-analysis of large phylogenies. Bioinformatics 30(9): 1312–1313. 10.1093/bioinformatics/btu033 PMC3998144 24451623

[B46] Swofford DL (2003) PAUP*: Phylogenetic analysis using parsimony (and other methods), version 4. Sunderland, Sinauer Associates, Sunderland, MA.

[B47] Thomas RJ (1987) Distribution of *Termitomyces heimi* and other fungi in the nests and major workers of *Macrotermes bellicosus* in Nigeria. Soil Biology and Biochemistry 19(3): 329–333. 10.1016/0038-0717(87)90018-6

[B48] Visser AA, Ros VID, De Beer ZW, Debets AJM, Hartog E, Kuyper TW, Læssøe T, Slippers B, Aanen DK (2009) Levels of specificity of *Xylaria* species associated with fungus‐growing termites: a phylogenetic approach. Molecular Ecology 18(3): 553–567. 10.1111/j.1365-294X.2008.04036.x 19161474

[B49] Wangsawat N, Ju YM, Phosri C, Whalley AJ, Suwannasai N (2021) Twelve new taxa of *Xylaria* associated with termite nests and soil from northeast Thailand. Biology 10(7): 575. 10.3390/biology10070575 PMC8301132 34201676

[B50] Westphal KR, Heidelbach S, Zeuner EJ, Riisgaard-Jensen M, Nielsen ME, Vestergaard SZ, Bekker NS, Skovmark J, Olesen CK, Thomsen KH, Niebling SK, Sørensen JL, Sondergaard TE (2021) The effects of different potato dextrose agar media on secondary metabolite production in *Fusarium*. International Journal of Food Microbiology 347: 109171. 10.1016/j.ijfoodmicro.2021.109171 33872940

[B51] White TJ, Bruns T, Lee S, Taylor J (1990) Amplification and direct sequencing of fungal ribosomal RNA genes for phylogenetics. In: Innis MA, Gelfand DH, Sninsky JJ, White TJ (Eds) PCR Protocols: A Guide to Methods and Applications. Academic Press, New York, 315–322. 10.1016/B978-0-12-372180-8.50042-1

[B52] Xing HL, Zhao YH, Lin F, Zhu WH, Wang CL (2002) Determination of total alkaloid content in *Sophora alopecuroides*. Journal of Pharmaceutical Practice of Chinese People’s Liberation Army 18(2): 113–115. [in Chinese]

[B53] Yang EF, Bodawatta KH, Tibpromma S, Samarakoon MC, Rapior S, Chiu CI, Han LS, Feng J, Meegaskumbura M, Zhao ZX, Poulsen M, Karunarathna SC, Promputtha I (2025) Host and microbial defenses against fungal invaders in a fungus‐farming termite. Insect Science [early view]. 10.1111/1744-7917.70207 41403232

[B54] Ying JZ, Mao XL, Ma QM, Zong YC, Wen HA (1987) Icons of Medicinal Fungi from China. Science Press, Beijing, 575 pp.

[B55] Zhaxybayeva O, Gogarten JP (2002) Bootstrap, Bayesian probability and maximum likelihood mapping: exploring new tools for comparative genome analyses. BMC Genomics 3(1): 4. 10.1186/1471-2164-3-4 PMC100357 11918828

[B56] Zhou Y, Danbolt NC (2014) Glutamate as a neurotransmitter in the healthy brain. Journal of Neural Transmission 121(8): 799–817. 10.1007/s00702-014-1180-8 PMC4133642 24578174

