# Supplementary material for: Cultivation and preliminary chemical analyses of the sclerotium-forming, termite fungus garden-associated Xylaria minima
Source: MycoKeys. 2026 Aug 21;139:49–72. doi: 10.3897/mycokeys.139.206447 (PMC13525401; doi:10.3897/mycokeys.139.206447)
Supplement: Supplementary file 1 [file mycokeys-139-049-s001.docx]

**Supplementary Figures**

**Figure S1**. *Xylaria minima*. GMBCC2428 (a-c) Sclerotized hyphae formed on the surface of sclerotia after three months on CLA plate; (d) Sclerotized hyphae observed under the microscope; (e-h) stromatal surface bearing upright conidiophores, with conidiogenous cells producing conidia stained with Congo red or cotton blue. (i,j) Conidia stained with cotton blue.

**Figure S2**. Sclerotia formed on two-gram pieces of mature fungus comb incubated in plastic boxes for two weeks. (A) Sclerotia forming on the surface of comb pieces; (B) Sclerotia forming beneath the comb pieces or within their galleries

**FigureS3**. The sclerotium-forming *Xylaria* strain Mg-M4 growing on PDA, WA, and different concentrations of termite-comb leachate agar and its radial growth rates on the corresponding media on day 15.

**Figure S4**. (a-c) Examples of large sclerotia formed by the aggregation of numerous smaller sclerotia growing in clusters on Paper-Comb Substrates (PCS) after 15 days of cultivation. (d) Fresh sclerotia harvested from PCS after one month of cultivation. (e) Naturally dried sclerotia.
