# Supplementary material for: Cultivation and preliminary chemical analyses of the sclerotium-forming, termite fungus garden-associated Xylaria minima
Source: MycoKeys. 2026 Aug 21;139:49–72. doi: 10.3897/mycokeys.139.206447 (PMC13525401; doi:10.3897/mycokeys.139.206447)
Supplement: Supplementary file 2 [file mycokeys-139-049-s002.docx]

**Supplementary Tables**

**Table S1**. PCR conditions and primers used for amplification of *Xylaria* species

| **Locus** | **Primer** | **Primer DNA sequence (5'-3')** | **Annealing Temp.** | **References** |
| --- | --- | --- | --- | --- |
| ITS | ITS4 | TCCTCCGCTTATTGATATGC | 55 °C, 50 s | White et al., 1990 |
|  | ITS5 | GGAAGTAAAAGTCGTAACAAGG |  |  |
| ACT | 512F | ATGTGCAAGGCCGGTTTCGC | 56 °C, 50 s | Carbone & Kohn, 1999 |
|  | 738R | TACGAGTCCTTCTGGCCCAT |  |  |
| TUB2 | T1 | AACATGCGTGAGATTGTAAGT | 52 °C, 50 s | O’Donnell & Cigelnik, 1997 |
|  | T22 | TCTGGATGTTGTTGGGAATCC |  |  |
| RPB2 | 5F | GAY GAY MGW GAT CAY TTY GG | 57 °C, 55 s | Liu et al., 1999 |
|  | 7cR | CCC ATR TCC TTG TTR CCC AT |  |  |

**Table S2**. Species names, strain numbers, and corresponding GenBank accession numbers of the *Xylaria* taxa (TE) were used in the phylogenetic analyses. The newly generated sequences are in red, type strains are in bold, and “/” represents the unavailable sequences.

| **Taxon** | **Specimens** | **ITS** | **ACT** | **RPB2** | **TUB2** |
| --- | --- | --- | --- | --- | --- |
| *Podosordaria leporina* | YMJ2269 | PV098402 | PV207614 | PV207622 | PV207618 |
| ***Podosordaria muli*** | **WSP 167** | **NR_158883** | **GQ455450** | **GQ853038** | **GQ844839** |
| ***Xylaria acuminatilongissima*** | **HAST 623** | **EU178738** | **GQ853046** | **GQ853028** | **GQ502711** |
| *Xylaria angulosa* | HAST146296 | PQ582212 | PQ605193 | PQ605188 | PQ605183 |
| *Xylaria angulosa* | HAST146295 | PQ582213 | PQ605194 | PQ605189 | PQ605184 |
| ***Xylaria atrodivaricata*** | **HAST 95052001** | **EU178739** | **GQ853048** | **GQ853030** | **GQ502713** |
| *Xylaria brevifurcata* | YMJ986 | OQ845481 | OQ818371 | OQ851525 | OQ818431 |
| ***Xylaria brevifurcata*** | **YMJ1381** | **OQ845482** | **OQ818372** | **OQ851526** | **OQ818432** |
| ***Xylaria brunneovinosa*** | **HAST 720** | **EU179862** | **GQ853041** | **GQ853023** | **GQ502706** |
| *Xylaria cf. escharoidea* | SWUF1735.1 | MT622792 | MW459200 | / | MW459227 |
| *Xylaria cf. escharoidea* | SWUF 1739.1 | MT622794 | MW459201 | / | MW459228 |
| *Xylaria cf. nigripes* | SWUF 1731.2 | MT622790 | MW459202 | / | MW459229 |
| *Xylaria cf. nigripes* | SWUF 1736.1 | MT622791 | MW459203 | / | / |
| *Xylaria chaiyaphumensis* | SWUF 16-11.4 | MT622776 | MW459213 | / | MW459236 |
| ***Xylaria cirrata*** | **HAST 664** | **EU179863** | **GQ853042** | **GQ853024** | **GQ502707** |
| *Xylaria conica* | SWUF18-4.3 | MT622786 | MW459223 | / | / |
| ***Xylaria conica*** | **SWUF18-4.4** | **MT622787** | **MW459224** | **/** | **MW459243** |
| ***Xylaria coprinicola*** | **YMJ 1145** | **HM585020** | **HM585017** | **HM585019** | **HM585018** |
| ***Xylaria escharoidea*** | **HAST 658** | **EU179864** | **GQ853044** | **GQ853026** | **GQ502709** |
| *Xylaria fimbriata* | HAST 491 | GU324753 | GQ853040 | GQ853022 | GQ502705 |
| ***Xylaria fulvescens*** | **SWUF17-27.2** | **MT622780** | **MW459218** | **/** | **MW459238** |
| *Xylaria furcata* | HAST 646 | GU324757 | GQ853050 | GQ853032 | GQ502715 |
| *Xylaria furcata* | YMJ956 | OQ842973 | OQ818389 | OQ851543 | OQ818449 |
| ***Xylaria furcatula*** | **YMJ1103** | **OQ845484** | **OQ818374** | **OQ851528** | **OQ818434** |
| *Xylaria furcatula* | YMJ1099 | OQ845483 | OQ818373 | OQ851527 | OQ818433 |
| ***Xylaria griseosepiacea*** | **HAST 641** | **EU179865** | **GQ853049** | **GQ853031** | **GQ502714** |
| *Xylaria guepini* | HD 20120101 | OP863307 | OP856890 | OP856891 | OP856892 |
| ***Xylaria hoehnelii*** | **YMJ643** | **OQ845489** | **OQ818379** | **OQ851533** | **OQ818439** |
| *Xylaria hoehnelii* | YMJ1159 | OQ845493 | OQ818383 | OQ851537 | OQ818443 |
| *Xylaria hoehnelii* | HAST 642 | GU324759 | GQ853054 | GQ853036 | GQ502719 |
| ***Xylaria insignifurcata*** | **YMJ650** | **GU324758** | **GQ853051** | **GQ853033** | **GQ502716** |
| *Xylaria insignifurcata* | YMJ1198 | OQ845497 | OQ818387 | OQ851541 | OQ818447 |
| ***Xylaria insolita*** | **HAST 990903011251** | **MN655979** | **MN656985** | **MN656981** | **MN656983** |
| ***Xylaria intraflava*** | **HAST 725** | **EU179866** | **GQ853053** | **GQ853035** | **GQ502718** |
| ***Xylaria iriomotensis*** | **NBRC 33288** | **AB274815** | **PQ605195** | **PQ605190** | **PQ605185** |
| ***Xylaria ischnostroma*** | **SWUF18-22.1** | **MT622788** | **MW459225** | **/** | **MW459244** |
| ***Xylaria margaretae*** | **SWUF17-34.1** | **MT622778** | **MW459216** | **/** | **/** |
| *Xylaria margaretae* | SWUF 1734.2 | MT622779 | MW459217 | / | / |
| ***Xylaria mianyangensis*** | **HAST 145687** | **MZ888986** | **MZ901347** | **MZ901333** | **MZ901319** |
| *Xylaria mianyangensis* | WLS 2062 | MZ888993 | MZ901354 | MZ901340 | MZ901326 |
| *Xylaria mianyangensis* | WLS 2069 | MZ888994 | MZ901355 | MZ901341 | MZ901327 |
| *Xylaria mianyangensis* | WLS2077 | MZ888995 | MZ901356 | MZ901342 | MZ901328 |
| ***Xylaria minima*** | **SWUF18-3.2** | **MT622789** | **MW459226** | **/** | **MW459245** |
| *Xylaria minima* | Mg-M4 (GMBCC2428) | PX699341 | PX725335 | PX725347 | PX725359 |
| *Xylaria minima* | Mg-F1  (GMBCC2429) | PX699342 | PX725336 | PX725348 | PX725360 |
| *Xylaria minima* | Mg-M6  (GMBCC2430) | PX699343 | PX725337 | PX725349 | PX725361 |
| *Xylaria minima* | Mg-F2 (GMBCC2431) | PX699344 | PX725338 | PX725350 | PX725362 |
| *Xylaria minima* | Mg-M11 (GMBCC2432) | PX699345 | PX725339 | PX725351 | PX725363 |
| *Xylaria minima* | Mg-M12  (GMBCC2433) | PX699346 | PX725340 | PX725352 | PX725364 |
| *Xylaria minima* | Ma-M1  (GMBCC2434) | PX699347 | PX725341 | PX725353 | PX725365 |
| *Xylaria minima* | Ma-M3  (GMBCC2435) | PX699348 | PX725342 | PX725354 | PX725366 |
| *Xylaria minima* | Ma-M5  (GMBCC2436) | PX699349 | PX725343 | PX725355 | PX725367 |
| *Xylaria minima* | Ma-M7  (GMBCC2437) | PX699350 | PX725344 | PX725356 | PX725368 |
| *Xylaria minima* | Ma-M9  (GMBCC2438) | PX699351 | PX725345 | PX725357 | PX725369 |
| *Xylaria minima* | Ma-M11  (GMBCC2439) | PX699352 | PX725346 | PX725358 | PX725370 |
| ***Xylaria neonigripes*** | **HAST145669** | **MZ888988** | **MZ901349** | **MZ901335** | **MZ901321** |
| *Xylaria neonigripes* | WLS 2030 | MZ888987 | MZ901348 | MZ901334 | MZ901320 |
| *Xylaria neonigripes* | HAST 722 | GU324756 | GQ853047 | GQ853029 | GQ502712 |
| *Xylaria nigripes* | WLS2056 | MZ888989 | MZ901350 | MZ901336 | MZ901322 |
| *Xylaria nigripes* | YMJ653 | EU179868 | GQ853045 | GQ853027 | GQ502710 |
| ***Xylaria ochraceostroma*** | **HAST 401** | **EU179869** | **GQ853052** | **GQ853034** | **GQ502717** |
| ***Xylaria reinkingii var. microspora*** | **SWUF17-19.1** | **MT622769** | **MW459209** | **/** | **MW459234** |
| ***Xylaria ripicola*** | **KA11-0060-1** | **NR153251** | **/** | **KU554696** | **/** |
| *Xylaria ripicola* | KA11-0060-2 | KM817200 | / | KU554697 | / |
| ***Xylaria rogersionigripes*** | **HAST145684** | **MZ888991** | **MZ901352** | **MZ901338** | **MZ901324** |
| *Xylaria rogersionigripes* | WLS 2064 | MZ888990 | MZ901351 | MZ901337 | MZ901323 |
| ***Xylaria scoparia*** | **YMJ1435** | **OQ843014** | **OQ818429** | **OQ851583** | **OQ818489** |
| *Xylaria scoparia* | YMJ1428 | OQ843013 | OQ818428 | OQ851582 | OQ818488 |
| ***Xylaria siamensis*** | **SWUF17-20.2** | **MT622765** | **MW459208** | **/** | **MW459233** |
| ***Xylaria sihanonthii*** | **SWUF18-1.3** | **MT622785** | **MW459222** | **/** | **MW459242** |
| *Xylaria sihanonthii* | SWUF18-5.1 | MT622784 | MW459221 | / | MW459241 |
| ***Xylaria subescharoidea*** | **HAST 990604011188** | **MN655980** | **MN656986** | **MN656982** | **MN656984** |
| *Xylaria subescharoidea* | WLS 2026 | MZ888999 | MZ901360 | MZ901346 | MZ901332 |
| *Xylaria subescharoidea* | YMJ 1442 | MZ888997 | MZ901358 | MZ901344 | MZ901330 |
| *Xylaria subintraflava* | SWUF 1722.2 | MT622764 | MW459206 | / | MW459231 |
| *Xylaria subintraflava* | SWUF 1724.2 | MT622757 | MW459207 | / | MW459232 |
| ***Xylaria subintraflava*** | **SWUF16-4.3** | **MT622762** | **MW459204** | **/** | **MW459230** |
| ***Xylaria tenellifurcata*** | **YMJ1070** | **OQ845498** | **OQ818388** | **OQ851542** | **OQ818448** |
| *Xylaria tentaculata* | KA13-1324 | KM077163 | / | / | / |
| *Xylaria tentaculata* | KA13-1325 | KM077164 | / | / | / |
| ***Xylaria termiticola*** | **NBRC102095** | **AB274813** | **PQ605196** | **PQ605191** | **PQ605186** |
| ***Xylaria terricola*** | **YMJ 1375** | **MF577042** | **MF577045** | **MF577043** | **MF577044** |
| ***Xylaria theinhirunae*** | **SWUF17-44.1** | **MT622771** | **OQ845432** | **OQ851592** | **OQ845440** |
| *Xylaria theinhirunae* | SWUF16-10.1 | MT622773 | OQ845431 | OQ851591 | OQ845439 |
| *Xylaria thienhirunae* | SWUF16-6.2 | MT622770 | MW459210 | / | MW459235 |
| *Xylaria vinacea* | SWUF 182.1 | MT622781 | MW459219 | / | MW459239 |
| ***Xylaria vinacea*** | **SWUF18-2.10** | **MT622783** | **MW459220** | **/** | **MW459240** |

**Table S3**. The measured data of size and wet weight of sclerotia produced on fungus gardens of *Macrotermes t*ermites.

| **Colony** | **Termite fungus gardens** | **Size (mm)/wet weight (mg)** | | | | |
| --- | --- | --- | --- | --- | --- | --- |
| ***M. annandalei* C1** | Mature gardens | 5.5 × 4/68.2 | 3 × 3/29.5 | 10 × 6/75.3 | 5.5 × 4/27.8 | / |
|  | Fresh gardens | / | / | / | / | / |
| M. ***annandalei* C2** | Mature gardens | 3.5 × 3/33.1 | 4 × 4/39.1 | 4.5 × 3/18.6 | / | / |
|  | Fresh gardens | / | / | / | / | / |
| M. ***annandalei* C3** | Mature gardens | 4 × 3/23.3 | 3.5 × 3/16.8 | 4 × 3/18.9 | 4 × 3.5/18.2 | / |
|  | Fresh gardens | / | / | / | / | / |
| ***M. gilvus* C1** | Mature gardens | 6 × 5.5/93.8 | 7 × 6/109.2 | 6 × 5/82.2 | 8 × 6.5/162.8 | 6 × 5.5/54.2 |
|  | Fresh gardens | 3 × 1.5/43.6 | / | / | / |  |
| ***M. gilvus* C2** | Mature gardens | 6 × 4.5/107.4 | 6.5 × 5/103 | 6 × 5/95 | 3 × 2.5/67 | 6.5 × 6.5/59 |
|  | Fresh gardens | 5 × 3.5/91.5 | / | / | / |  |
| ***M. gilvus* C3** | Mature gardens | 5.5 × 5/58.7 | 5 × 4/45.1 | / | / |  |
|  | Fresh gardens | / | / | / | / |  |

**Table S4**. The BLASTn results of *Xylaria* spp. strains present in boxes formed sclerotia during the same period.

| ***S*trian** | **Accession No.** | **The high similarity in BLASTn searches** |
| --- | --- | --- |
| *Xyalria* sp. b2 | PX699353 | 99.81%: *Xylaria* sp. isolate X2024 |
| *Xyalria* sp. b3 | PX699340 | 99.78%: *Xylaria minima* isolate SWUF18-3.2 |
| *Xyalria* sp. b3-1 | PX699354 | 99.39%: *Xylaria ochraceostroma* voucher FCATAS3576 |
| *Xyalria* sp. c8 | PX699355 | 99.61%: *Xylaria reinkingii var. microspora* isolate SWUF17-19.1 |
